# Dual Role of Natural Killer Cells in Early Pregnancy: Immunopathological Implications and Therapeutic Potential in Recurrent Spontaneous Abortion and Recurrent Implantation Failure

**DOI:** 10.1111/cpr.70037

**Published:** 2025-05-05

**Authors:** Defeng Guan, Zhou Chen, Yuhua Zhang, Wenjie Sun, Lifei Li, Xia Huang

**Affiliations:** ^1^ The First Clinical Medical College, Lanzhou University Lanzhou Gansu China; ^2^ The First Hospital of Lanzhou University Lanzhou Gansu China; ^3^ Gansu Provincial Hospital Lanzhou Gansu China; ^4^ The Third Clinical Medical College, Lanzhou University Lanzhou Gansu China

**Keywords:** molecular mechanisms, natural killer cell, recurrent implantation failure, recurrent spontaneous abortion, targeted therapy

## Abstract

Natural killer (NK) cells are critical regulators of immune processes during early pregnancy, playing a key role in maintaining maternal‐foetal immune tolerance and supporting successful implantation. In particular, uterine NK cells, a specialised subset of NK cells, facilitate trophoblast invasion, spiral artery remodelling and placental establishment. Dysregulation of NK cell activity, however, has been implicated in pregnancy complications, notably recurrent spontaneous abortion (RSA) and recurrent implantation failure (RIF). Aberrant NK cell functions, such as heightened cytotoxicity or defective immune signalling, can disrupt the balance between immune tolerance and response, leading to impaired placental development, reduced trophoblast activity and compromised uteroplacental blood flow. This review examines the role of NK cells in early pregnancy, emphasising their contributions to immune modulation and placentation. It also investigates the mechanisms by which NK cell dysfunction contributes to RSA and RIF, and explores therapeutic strategies aimed at restoring NK cell balance to improve pregnancy outcomes. A deeper understanding of NK cell interactions during early pregnancy may provide critical insights into the pathogenesis of pregnancy failure and facilitate targeted immunotherapeutic approaches.

## Graphical abstract

This article elaborates on the role of NK cells in early pregnancy from multiple perspectives, including molecular mechanisms and therapeutic interventions. It also discusses how NK cell dysfunction contributes to RSA and RIF, aiming to provide insights for future research on NK cell mechanisms and clinical treatment of RSA and RIF.

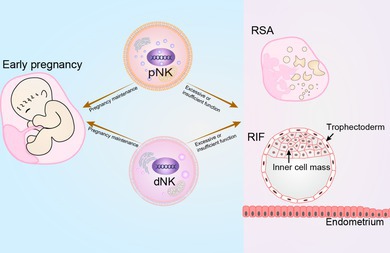

## 1 Introduction

In early pregnancy, immune cells play a crucial role at the maternal‐foetal interface, with natural killer (NK) cells being particularly important [1, 2]. These cells contribute to immune defence and play a key role in maintaining pregnancy and facilitating placental development. Based on their origin and function, NK cells can be categorised into peripheral NK (pNK) and uterine NK (uNK) cells [3]. When uNK cells migrate to the decidua, they gradually differentiate into decidual NK (dNK) cells with distinct functional characteristics [4]. dNK cells are a pregnancy‐specific subset of NK cells predominantly observed in the decidual tissue, accounting for 50%–70% of decidual immune cell [5].

dNK cells and pNK cells exhibit notable phenotypic and functional differences. dNK cells demonstrate lower cytotoxicity and enhanced secretory activity, particularly in the secretion of cytokines such as interferon‐γ (IFN‐γ), vascular endothelial growth factor (VEGF) and placental growth factor (PLGF), which play crucial roles in pregnancy‐specific processes [6, 7, 8]. These cytokines are essential for placental vascular remodelling, trophoblast invasion and the maintenance of placental immune tolerance, all of which are vital for appropriate placental development and normal foetal growth.

However, dysregulation of dNK cell function is typically associated with pregnancy failure. Overactivation of dNK cells may result in excessive cytotoxicity, leading to damage to trophoblast cells and impaired placental development, which in turn can trigger recurrent spontaneous abortion (RSA) and recurrent implantation failure (RIF) [9]. Conversely, insufficient dNK cell function or abnormal cytokine secretion, such as reduced levels of IFN‐γ and VEGF, can result in incomplete vascular remodelling and inadequate placental blood supply, increasing the risk of pregnancy failure or foetal growth restriction. Additionally, imbalances in the interactions between dNK cells and other immune cells in the decidua, such as macrophages and regulatory T cells (Tregs), may disrupt the maternal‐foetal immune tolerance environment, further increasing the risk of pregnancy‐related disorders [10].

The interactions between dNK cells and other immune cells, such as Tregs, are crucial for immune regulation. Disruptions in the balance of these cellular functions can cause immune rejection and further increase the risk of pregnancy failure [11, 12]. Therefore, a deeper understanding of the functional regulation of dNK cells and their interactions with other immune cells is essential for elucidating the pathological mechanisms of pregnancy failure and developing targeted therapeutic strategies.

## 2 Early Pregnancy NK Cells

**FIGURE 1:**
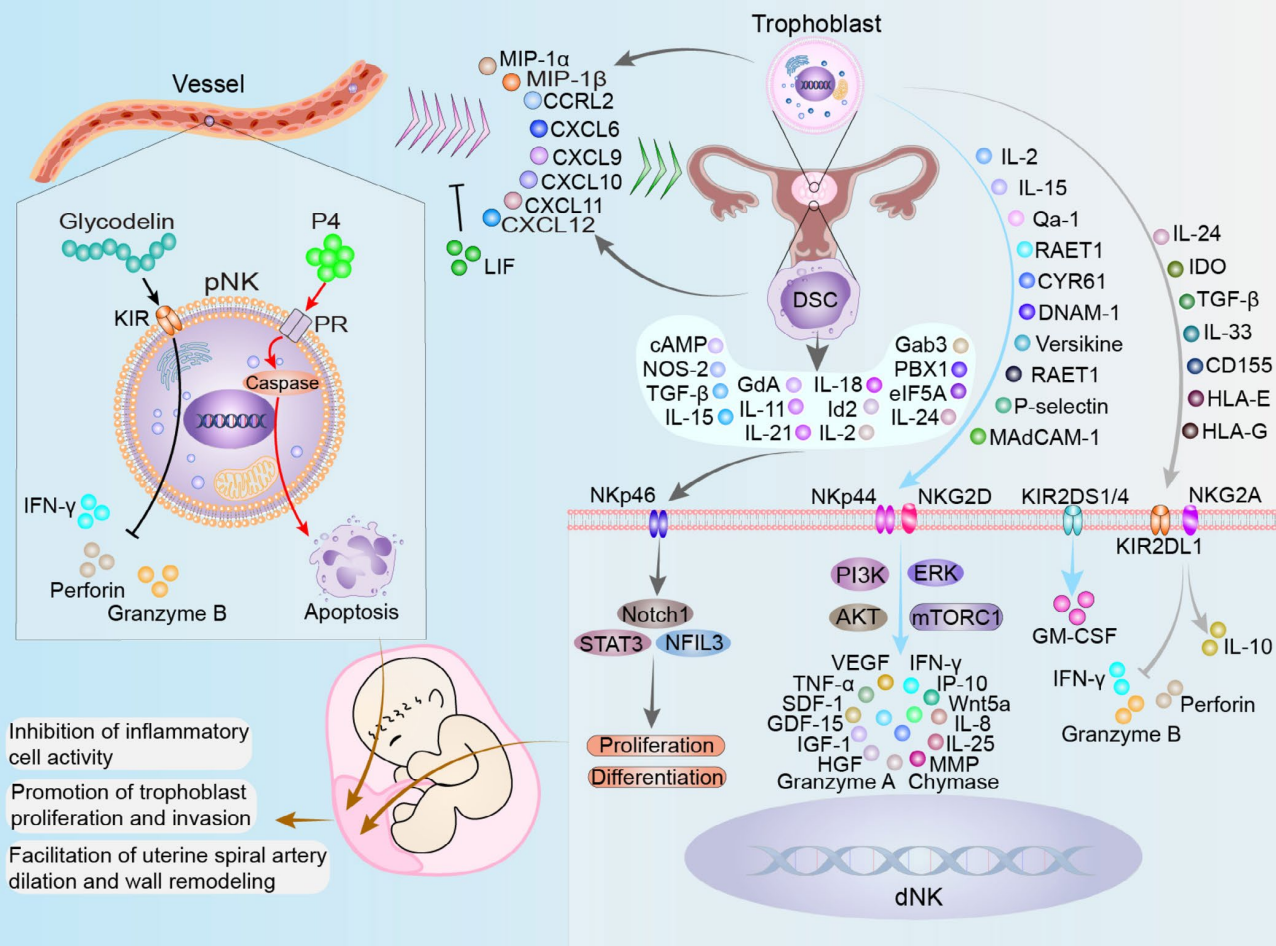
Role of NK cells in normal pregnancy. NK cells are essential in normal pregnancy, balancing immune tolerance and tissue remodelling. Glycodelin inhibits pNK cell cytotoxicity by downregulating perforin, granzyme B and IFN‐γ through KIR receptors, while progesterone induces caspase‐dependent pNK cell apoptosis via PR. Trophoblasts and ESCs recruit pNK cells by expressing chemokines, including MIP‐1α, MIP‐1β, CCRL2, CXCL6, CXCL9, CXCL10, CXCL11 and CXCL12, with LIF limiting excessive NK cell migration to the uterus. Factors secreted by DSCs, such as cAMP, NOS‐2, TGF‐β, IL‐15, GdA and IL‐24, promote dNK cell proliferation and differentiation by activating pathways like STAT3, NFIL3 and Notch1 via NKp46. Trophoblast‐secreted factors, including IL‐2, IL‐15, RAET1, DNAM‐1 and P‐selectin, activate pathways such as ERK, mTORC1, PI3K and AKT through NKp44 and NKG2D, inducing dNK cells to secrete VEGF, IL‐8, IFN‐γ, TNF‐α, and growth factors that support trophoblast proliferation, uterine spiral artery remodelling, and vascular dilation. Additionally, trophoblast‐derived IL‐24, IDO, TGF‐β, IL‐33, HLA‐E and HLA‐G interact with inhibitory receptors like NKG2A and KIR2DL1, suppressing perforin and granzyme B expression, enhancing IL‐10 secretion and maintaining immune tolerance. Activation of KIR2DS1/4 supports GM‐CSF secretion by dNK cells, collectively inhibiting inflammatory cell activity, promoting trophoblast proliferation and invasion, and facilitating uterine vascular remodelling.

During early pregnancy, pNK and uNK cells undergo distinct changes, reflecting the adaptive processes of the maternal immune system to support foetal development. These changes involve both phenotypic and functional modifications that enable the immune system to balance immune defences for immune tolerance of the developing foetus. pNK cells primarily function in immune surveillance and defence; however, when they migrate to the uterus, uNK cells play critical roles in facilitating implantation, promoting trophoblast invasion and establishing placental blood flow, all of which are essential for a successful pregnancy (Figure 1).

### 2.1 pNK Cells

During early pregnancy, the number of maternal pNK cells typically decreases. Although pNK cells continue to play an important role in the immune response during early pregnancy, their numbers are lower than those in non‐pregnant women. pNK cells express two classical progesterone receptor (PR) subtypes and are influenced by progesterone (P4) through two seemingly independent mechanisms. Progesterone induces caspase‐dependent death of pNK cells [13].

Killer immunoglobulin‐like receptors (KIRs) are a class of immunoglobulin superfamily receptors on the surface of NK cells [14]. By binding to ligands on the target cell surface (mainly major histocompatibility complex (MHC) class I molecules), KIRs determine whether NK cells are activated or inhibited. These receptors include inhibitory KIRs (such as KIR2DL and KIR3DL3), which contain inhibitory signalling structures [15]. When these receptors bind to MHC‐I molecules on the cell surface, they send inhibitory signals that suppress NK cell activation. Inhibitory KIRs prevent the immune system from erroneously attacking healthy cells by inhibiting NK cell cytotoxicity and cytokine secretion [16, 17]. In contrast, activating KIRs (such as KIR2DS and KIR3DS) have the opposite effect; they promote NK cell activation by recognising specific ligands or changes in MHC‐I molecules, thereby enhancing NK cell cytotoxicity [18, 19].

Glycodelin, a glycoprotein, induces apoptosis in NK cells [20]. Glycodelin inhibits the cytotoxicity of pNK cells by downregulating perforin, granzyme B and IFN‐γ [5]. Compared to normal non‐pregnant controls, the relative activity of pNK cells increases during the first 3 months of pregnancy [21]. In early pregnancy, immune tolerance is gradually established to prevent maternal immune rejection of the foetus. During this time, the cytotoxic activity of pNK cells decreases, and their immune response is regulated. However, specific mechanisms underlying this regulation remain unclear.

### 2.2 Proliferation and Differentiation of dNK Cells

pNK cells and cord blood NK cells share highly similar immune phenotypes and functional characteristics [22]. However, early pregnancy dNK cells possess unique features that distinguish them significantly from pNK cells [23]. In early pregnancy, particularly after embryo implantation, the number of dNK cells increases significantly, accounting for 70%–80% of the immune cells in the uterine decidua [24]. Typically, uNK cells are recruited from peripheral blood and undergo further differentiation in the microenvironment at the maternal‐foetal interface, resulting in the development of a dNK‐like phenotype [25, 26].

This process is facilitated by the upregulation of several chemokines during decidualization, such as C‐C chemokine receptor‐like 2 (CCRL2) [27], monocyte inflammatory protein‐1α (MIP‐1α) [28], MIP‐1β [29], CXCL6 [30], CXCL12 [31, 32], CXCL9, CXCL10 and CXCL11 [33, 34]. The expression of these chemokines is possibly associated with an increase in progesterone levels [2]. To maintain balance, leukaemia inhibitory factor (LIF) restricts the migration of NK cells to the uterus [35]. Interestingly, mature NK cells do not migrate into the decidua or uterus. Instead, precursors are recruited to these organs, where they differentiate into NK cells [36].

The recruited dNK cells are stimulated by cytokines enriched in the decidual microenvironment, resulting in their development and differentiation into functionally distinct subpopulations. This transition is associated with a decrease in cytotoxic function and a significant adaptation in the secretory profile. During the decidualisation process, cyclic adenosine monophosphate (cAMP) plays a key role in efficient decidualisation. cAMP is produced by decidualised endometrial stromal cells (ESCs), leading to its accumulation at the maternal–fetal interface [37]. cAMP activates downstream pathways and induces the expression and nuclear localisation of forkhead box protein O1 (FOXO1). Activation of FOXO1 upregulates the expression of CD56 on dNK cells recruited from the peripheral blood, resulting in a predominance of CD56^bright^ NK cells in the decidua. CD56^bright^ NK cells are characterised by relatively low cytotoxicity and high cytokine production ability [38].

Another key cytokine driving dNK cell proliferation and development is interleukin (IL)‐15, which is primarily derived from dendritic cells (DCs) and macrophages. IL‐15 plays a crucial role in promoting the proliferation and development of dNK cells [39, 40, 41]. IL‐15 also facilitates the initial interaction between pNK cells and human uterine microvascular endothelial cells [42]. The absence or blockade of IL‐15 inhibits NK cell differentiation and impairs their function [43]. The inhibitor of DNA binding 2 (Id2), an antagonist of E protein transcription factors, binds to E proteins and prevents their association with E box sequences in DNA. Id2 is essential for the development of mature NK cells [44, 45]. By inhibiting multiple E protein target genes, including Socs3, Id2 plays a critical role in regulating IL‐15 receptor signalling and maintaining NK cell homeostasis [46, 47]. Furthermore, Id2 cooperates with IL‐15 to promote NK cell expansion and differentiation [48, 49].

Other studies have shown that IL‐2 [50], IL‐11 [51], IL‐18 [52], IL‐21 [53], IL‐24 [54], transforming growth factor‐beta (TGF‐β) [52, 55], pre‐B‐cell leukaemia transcription factor 1 [56, 57], Grb2‐associated binding protein 3 [58], NO synthase [59] and eukaryotic initiation factor 5A (eIF5A) [60] activate dNK cell surface receptors, such as natural cytotoxicity receptor‐1 (NKp46) [61] and promote dNK cell proliferation, which is essential for their differentiation. This process is mechanistically linked to the activation of STAT3 [53], IL‐3‐regulated protein (NFIL3) [56, 62, 63] and Notch1 [64].

Glycodelin‐A (GdA), a glycoprotein abundant in human decidua, reaches peak expression between weeks 6 and 12 of pregnancy and stimulates the transformation of human peripheral blood CD16^−^ CD56^bright^ NK cells into a dNK cell‐like phenotype [65]. Mass spectrometry flow cytometry analysis has categorised early pregnancy dNK cells into three subpopulations: dNK1, dNK2 and dNK3. After stimulation, chemokine production from dNK2 and dNK3 was significantly higher than that from dNK1, including the chemokine XCL1. In contrast, dNK1 cells express receptors such as KIRs and are associated with higher levels of granzyme B [66].

Additionally, some key low‐toxicity NK cell subsets have been identified, such as Cytochrome P450 26A1 (CYP26A1) cells, which affect embryo implantation by modulating NK cells. Knockdown of CYP26A1 in NK cells significantly upregulates the expression of granzyme A and NKG2D, whereas the expression of VEGF‐C, SLAM family member 6 and Fc gamma receptor IV (FCGR4) is reduced [7, 67, 68]. These findings suggest a model of NK cell proliferation and differentiation that will enhance our understanding of their function during early pregnancy [69].

### 2.3 Functions of dNK Cells

Trophoblast cells express a variety of molecules, including DNAX accessory molecule 1 (DNAM‐1) [70], retinoic acid early transcript 1 (RAET1) [71], Qa‐1 [72], IL‐2 and IL‐15 [73], cysteine‐rich angiogenic inducer 61 [74], mucosal addressin cell adhesion molecule‐1 (MAdCAM‐1) and P‐selectin [75] and versikine [76]. These molecules activate receptors such as NKG2D [71] and NKp44 [70] and induce the activation of signalling pathways, including phosphorylation of ERK 1/2 [77], mechanistic target of rapamycin complex 1 (mTORC1) [72] and the phosphatidylinositol 3 kinase (PI3K)/protein kinase B (AKT) signalling pathway [74]. This activation stimulates dNK cells to release a range of factors, including VEGF, stromal cell‐derived factor‐1, IFN‐γ‐inducible protein‐10 (IP‐10), IL‐8, IFN‐γ, granzyme A, PLGF and tumour necrosis factor‐alpha (TNF‐α) [78, 79]. Building on these molecular interactions and signalling pathways, dNK cells undergo morphological changes, including active proliferation, an increasing number of cytoplasmic granules, and an enlarged cell diameter [80, 81, 82], while establishing close associations with stromal cells at the ultrastructural level to play a pivotal role in pregnancy‐related angiogenesis and decidualization [83, 84]. These functions mainly include immune regulation, promotion of trophoblast cell proliferation and invasion, remodelling of uterine spiral arteries and promotion of trophoblast cell angiogenesis.

dNK cells contribute to immune regulation by secreting IFN‐γ, which suppresses inflammatory cells such as Th17 cells and DCs, promoting immune tolerance and supporting a successful pregnancy [85, 86]. Additionally, excessive immune activation can negatively impact pregnancy outcomes. For instance, IFN‐γ significantly increases uterine CX3CL1 expression by activating the JAK2‐STAT1 pathway, which recruits CD49b^+^ NK cells to the uterus, potentially causing foetal loss [87]. Studies have shown a reduced production of IFN‐γ by uNK cells during early pregnancy, which might be associated with complement C5a's role in suppressing excessive immune responses [1, 88, 89].

dNK cells enhance trophoblast cell proliferation and invasion through various mechanisms. They secrete insulin‐like growth factor‐1 (IGF‐1) and growth differentiation factor‐15, which facilitate trophoblast invasion and embryonic growth [90]. IGF‐1 binding to IGF‐1R reduces apoptosis in decidual stromal cells (DSCs) and downregulates dNK cytotoxicity [91]. Additionally, dNK cells express wingless ligand 5a, activating the MAPK pathway to promote the proliferation of villous and columnar trophoblasts [92].

dNK cells play a crucial role in remodelling uterine spiral arteries during early pregnancy. They facilitate the loss of smooth muscle cells and subendothelial elastic fibres in these arteries, resulting in thinner, low‐resistance, high‐capacitance vessels. This vascular remodelling improves placental oxygen and nutrient supply while protecting placental tissues from high‐pressure maternal blood flow [93]. These processes are mediated by factors released by dNK cells, contributing to successful pregnancy.

dNK cells promote trophoblast cell angiogenesis by secreting factors such as VEGF and hepatocyte growth factor (HGF) [94]. These factors stimulate trophoblast differentiation into an endothelial phenotype, enhancing the formation of capillaries and vascular networks [95]. Moreover, dNK cells demonstrate a greater capacity than umbilical cord blood NK cells or pNK cells in inducing human umbilical vein endothelial cell formation [96]. Through the secretion of matrix metalloproteinases (MMP2 and MMP9), dNK cells degrade collagen, weaken vascular wall strength and allow trophoblast invasion into arterial lumens [97]. Through these actions, dNK cells support trophoblast cell angiogenesis and placental vascular development.

These cells are essential for immune tolerance and the regulation of placental vascular remodelling. The absence or dysfunction of dNK cells can result in decidual abnormalities, including arterial wall thickening, luminal narrowing and a reduction in basal decidual cells [98, 99].

### 2.4 Inhibition of dNK Cytotoxicity

The inhibition of cytotoxic receptors in decidual NK (dNK) cells is primarily attributed to changes in receptor expression and the presence of inhibitory factors produced by trophoblast cells. Studies have identified two major types of receptors on dNK cells: NKG2 and KIRs [100].

The NKG2 receptor family consists of C‐type lectin‐like membrane proteins, primarily expressed on NK cells and certain immune cells such as CD8+ T cells [101]. These receptors regulate NK cell activation or inhibition through specific ligand interactions and play crucial roles in immune surveillance, antiviral defence and tumour immunity [101]. Based on their functions, NKG2 receptors are classified into inhibitory receptors (NKG2A) and activating receptors (NKG2C and NKG2D) [102]. During early pregnancy, the proportion of NKG2A‐positive cells is significantly higher in decidual CD56bright NK cells compared to peripheral CD56dim NK cells [103]. When early pregnancy dNK cells are co‐cultured with trophoblast cell lines, NKG2D expression is markedly reduced [104]. Similarly, NKG2D expression is downregulated in maternal peripheral blood mononuclear cells and cord plasma [105]. Additionally, indoleamine 2,3‐dioxygenase (IDO) and soluble NKG2D ligands can suppress NKp46 and NKG2D expression, reducing the cytotoxicity of peripheral NK (pNK) cells. This mechanism may help maintain the low cytotoxicity of dNK cells while decreasing IFN‐γ secretion [106, 107]. Trophoblast cells express CD155, which activates CD96^+^ dNK cells, thereby inhibiting the secretion of cytotoxic factors such as IFN‐γ and granzyme B while upregulating inhibitory cytokines such as IL‐10. This process reduces the immunotoxicity of dNK cells [108]. Although activating receptors on dNK cells, such as NKp30, NKp44, NKp46 and NKG2D, are stimulated during early pregnancy, leading to increased expression of perforin, granzyme A and granzyme B, dNK cells differ from pNK cells in that they are unable to effectively polarise their microtubule‐organising centres and perforin‐containing granules to the immunological synapse. This limitation restrains their cytotoxicity and contributes to maintaining immune homeostasis at the maternal‐foetal interface during pregnancy [109, 110].

KIRs are expressed on the surface of NK cells and certain T cells, belonging to the immunoglobulin superfamily. These receptors interact with MHC I molecules, regulating NK cell activation and inhibition, and play crucial roles in infection control, tumour immunity and maternal‐foetal immune tolerance [111, 112]. Functionally, KIRs are categorised into inhibitory and activating receptors. Inhibitory KIRs, such as KIR2DL1, KIR2DL2 and KIR3DL1, recognise specific peptides presented by MHC I molecules and transmit inhibitory signals, preventing NK cells from attacking normal cells [15]. In contrast, activating KIRs, including KIR2DS1 and KIR3DS1, bind to specific MHC I molecules or other ligands, transmitting activation signals that promote NK cell‐mediated clearance of abnormal cells, such as tumour cells or virus‐infected cells [18]. In uNK cells, KIRs exhibit unique properties that enhance their recognition of trophoblast cells expressing human leukocyte antigen C (HLA‐C) at the maternal‐foetal interface [113]. The interaction between KIRs and HLA molecules directly influences pregnancy outcomes, with specific HLA‐KIR combinations regulating uNK cell activation levels. Insufficient activation has been associated with pregnancy complications such as preeclampsia, foetal growth restriction and recurrent miscarriage, highlighting the essential role of KIR‐mediated uNK activation in embryo implantation [114, 115, 116]. For example, KIR2DS1 and KIR2DS4 are highly expressed in uNK cells and strongly stimulate the secretion of granulocyte‐macrophage colony‐stimulating factor (GM‐CSF), which promotes trophoblast migration and invasion [117, 118]. However, the absence or insufficiency of inhibitory KIR ligands, such as KIR2DL2, may disrupt maternal uNK‐mediated inhibition of trophoblasts, leading to adverse pregnancy outcomes [119]. Additionally, inhibitory KIRs, including KIR2DL1 and KIR2DL3, contribute to reduced NK cell reactivity. This effect, together with TGF‐β‐induced suppression, helps modulate NK cell responses to target cells, ensuring immune tolerance and a successful pregnancy [120]. dNK cells express various inhibitory receptors, such as LAIR‐1 [121] and 2B4 [122]. In murine dNK cells, Ly49 receptors are also present, playing a crucial role in suppressing cytotoxicity and IFN‐γ production, thereby supporting immune regulation during pregnancy [123]. Ly49 receptors have not been identified in human NK cells, whereas their presence in mice suggests significant differences in receptor expression and functional regulation between the two species. The application of new technologies, such as advanced mouse models, high‐throughput genotyping, mass cytometry and single‐cell RNA sequencing, will help to more precisely elucidate the role of immune cells in pregnancy, particularly in understanding the functional differences between human and murine NK cells and their impact on pregnancy outcomes [124, 125].

During early pregnancy, placental trophoblast cells predominantly express nonclassical HLA molecules, including HLA‐C, HLA‐E and HLA‐G, while lacking classical HLA‐A and HLA‐B. These nonclassical HLA molecules interact with KIRs on maternal uNK cells, modulating immune responses to facilitate embryo implantation and placental development. The expression of class I HLA mRNA is induced by progesterone and increases during decidualization [126]. Through the interaction between HLA‐C and KIRs, uNK cell activity is finely regulated to maintain immune balance at the maternal‐foetal interface, supporting placental development, which is critical for a successful pregnancy [127]. HLA‐G plays a particularly significant role in regulating dNK cell function. Its interaction with specific receptors reduces the expression of signal transducer and activator of transcription 3 (STAT3) in dNK cells, thereby inhibiting perforin secretion and limiting dNK cytotoxicity [128]. Additionally, these interactions stimulate dNK cells to secrete angiogenic factors, further enhancing placental development [129]. HLA‐G also activates key signalling pathways, including DNA‐dependent protein kinase catalytic subunit (DNA‐PKcs), AKT and nuclear factor kappa‐B (NF‐κB), which induce pro‐inflammatory and pro‐angiogenic responses, as well as the senescence‐associated secretory phenotype (SASP). These processes contribute to growth arrest, tissue repair, vascular remodelling and angiogenesis, ultimately optimising reproductive capacity [130]. Circulating HLA‐G further supports NK cell tolerance, reinforcing immune homeostasis during pregnancy [131, 132].

Moreover, IL‐33 derived from DSCs modulates dNK cell function via the NF‐κB signalling pathway, inducing a Th2‐biased immune response while suppressing dNK cytotoxicity, thereby playing a crucial role in early pregnancy [133]. In summary, dNK cells contribute to the formation of a localised inflammatory environment during early pregnancy by secreting pro‐inflammatory factors such as TNF‐α, IFN‐γ and GM‐CSF, promoting decidualisation, embryo implantation and vascular remodelling [134]. Following successful implantation, dNK cells transition to an anti‐inflammatory phenotype, secreting factors such as IL‐10 and TGF‐β to maintain immune tolerance at the maternal‐foetal interface, ensuring pregnancy stability [135]. Dysregulation of dNK cell function is closely associated with adverse pregnancy outcomes.

## 3 RSA and RIF in Relation to NK Cells

RSA and RIF are the two most common adverse pregnancy outcomes. According to the latest definition by the European Society of Human Reproduction and Embryology, RSA refers to the loss of two or more pregnancies before 24 weeks of gestation, excluding ectopic and molar pregnancies [136]. RIF is the failure to achieve a successful pregnancy after multiple (typically ≥ 3) transfers of high‐quality embryos, primarily manifesting as an inability of the embryo to implant [137]. One of the underlying causes of RSA and RIF is closely related to the activity and function of NK cells.

### 3.1 RSA and NK Cells

#### 3.1.1 pNK Cells

**FIGURE 2:**
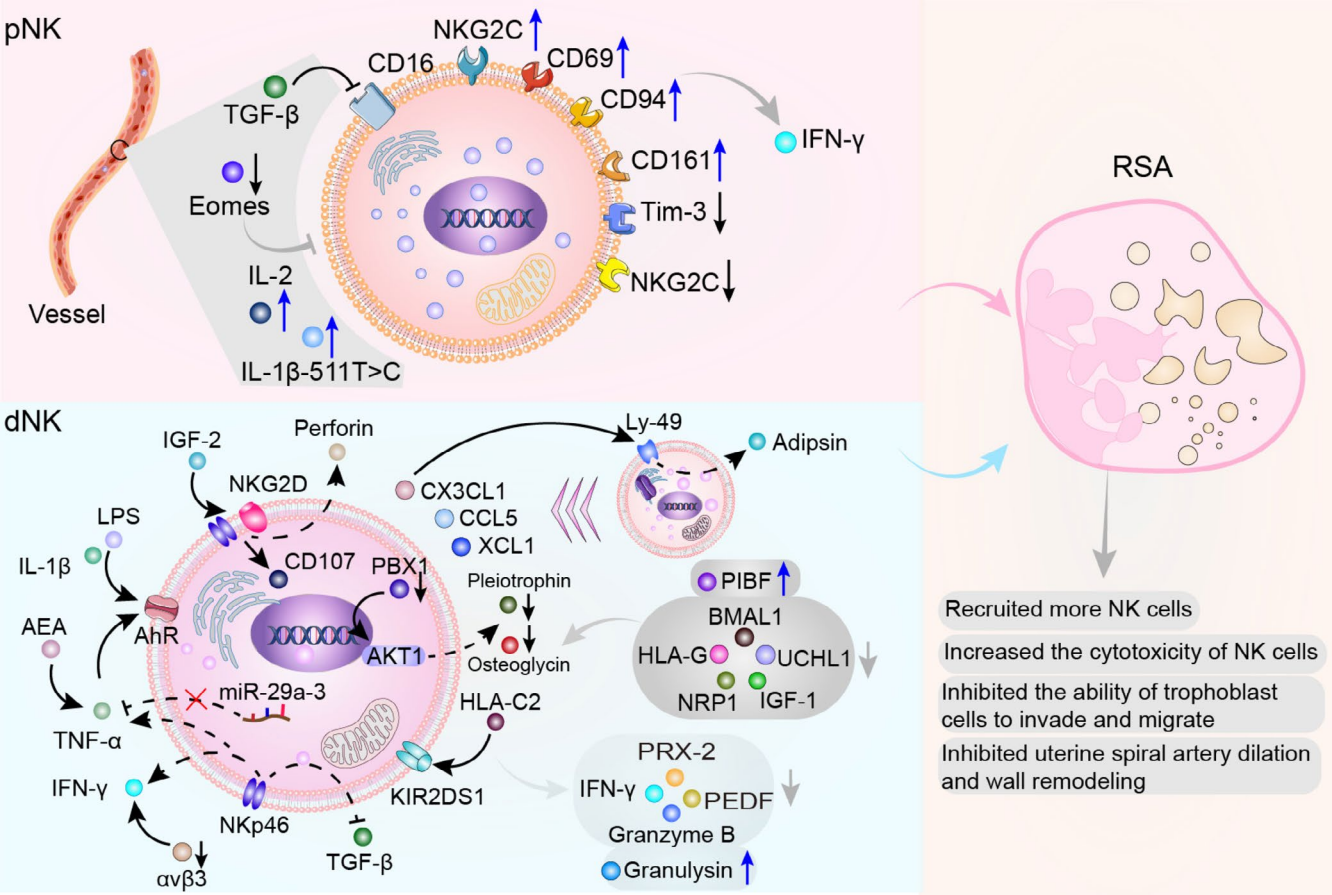
Role of NK cells in RSA. In RSA, CD56^dim^ NK cells increase, showing elevated expression of receptors like CD94, CD69, CD161 and NKG2C, alongside decreased inhibitory receptors such as LILRB1 and Tim‐3, leading to increased IFN‐γ secretion and cytotoxicity. TGF‐β in the blood inhibits pNK cells from acquiring CD16, thereby suppressing their differentiation. Eomes‐deficient mice fail to develop NK cells. Other factors, such as IL‐1β‐511T>C and IL‐2, enhance NK cell cytotoxicity and increase susceptibility to RSA. Chemokines like XCL1, CCL5 and CX3CL1 recruit cytotoxic CD56^dim^ CD16high NK cells, and CX3CL1 induces high Ly49 receptor expression in CD49b^+^ dNK cells. DSC autophagy inhibition increases IGF‐2 expression, activating dNK cells via NKG2D and NKp46, enhancing perforin release and inducing trophoblast apoptosis. TNF‐α, IL‐1β and LPS upregulate AhR in dNK cells, while elevated AEA levels induce TNF‐α secretion, exacerbating inflammation. Abnormal activating receptor expression, such as KIR2DS1 interacting with HLA‐C2 ligands, impairs pregnancy outcomes. The decrease in PBX1 expression in dNK cells leads to insufficient activation of AKT1, which results in inadequate expression of pleiotrophin and osteoglycin, thereby contributing to RSA. When HLA‐G expression in trophoblast cells, BMAL1 and UCHL1 expression in decidual cells, and IGF‐1 expression in stromal cells are insufficient, or NRP1 expression in DSCs is reduced, or PIBF levels are elevated, NK cell numbers increase, enhancing their cytotoxic activity. Decreased expression of IFN‐γ, Granzyme B, PRX‐2 and PEDF in NK cells, or elevated granulysin levels, can inhibit the invasive and migratory abilities of trophoblast cells, ultimately leading to RSA.

Between 4 and 6 weeks of pregnancy, pNK cell levels in patients with unexplained RSA show a significant decline compared to pre‐pregnancy levels. This decline may serve as an effective predictor of live birth rates [138]. However, compared to healthy pregnancies, the immune system of patients with RSA is highly active, with a marked increase in the proportion of pNK cells. Patients with RSA exhibit significantly higher overall expression levels of CD69, CD94, CD161 [139] and CD158b [140, 141, 142] (Figure 2). The proportion of CD56^dim^ NK cells, which primarily mediate cytotoxicity and possess strong killing activity, is significantly elevated in the peripheral blood of patients with RSA compared to healthy parous women [140, 141, 143, 144]. Interestingly, the proportion of CD56^bright^ NK cells is also higher in patients with RSA than controls. These CD56^bright^ NK cells upregulate NKp46, NKp44 and NKp30 through a2V‐ATPase activation. However, despite this compensatory activation mechanism, pregnancy outcomes remain unimproved [145].

TGF‐β in the bloodstream can convert CD56^bright^ CD16^+^ NK cells into CD56^bright^ CD16^−^ NK cells [146]. Mechanistically, the imbalance in pNK cell receptor expression is closely associated with adverse pregnancy outcomes. For example, the activation receptor NKG2C is significantly upregulated, whereas the inhibitory receptor LILRB1 is markedly reduced in the peripheral blood of patients with RSA [147]. This abnormal activation possibly causes excessive secretion of IFN‐γ by pNK cells, resulting in unfavourable pregnancy outcomes [148].

Furthermore, T‐cell immunoglobulin and mucin‐domain containing protein 3 (Tim‐3) signalling in pNK cells plays a crucial protective role during early pregnancy. During normal pregnancy, Tim‐3 expression in pNK cells increases transiently during the first trimester. Upon stimulation by its ligand Galectin‐9 (Gal‐9), Tim‐3 activates the JNK and AKT signalling pathways, resulting in the production of anti‐inflammatory cytokines and TGF‐β1, which suppress the cytotoxicity of pNK cells towards trophoblasts [149, 150, 151]. However, in patients with RSA, Tim‐3 expression on pNK cells is reduced, impairing their immunosuppressive function [152, 153]. IL‐1β–511T>C and increased IL‐2 levels act as potential triggers for RSA susceptibility [154, 155]. However, the significant reduction in pNK cell activity is also closely associated with RSA, although the exact mechanism remains unclear [156].

Additionally, NK cells in menstrual blood may reflect adverse pregnancy outcomes. Compared with healthy individuals, patients with RSA exhibit a significant decrease in CD45RO^+^ NK cells [157], CD49a^+^ Eomes^+^ NK subsets [158] and CD56^dim^CD16^bright^ NK cells [159] in their menstrual blood. CD49a^+^ Eomes^+^ NK cells are characterised by low toxicity and secrete growth‐promoting factors, including pleiotrophin and osteoglycin, which improve uterine arterial blood flow and promote foetal growth [160, 161]. Eomes deficiency in mice results in an inability to develop mature NK cells [162, 163, 164, 165, 166]. NFIL3 drives the maturation of NK cells by inducing the binding of Eomes and Id2 [167, 168].

However, other studies have found a higher frequency of CD49a^+^ NK cells in menstrual blood, which correlates with serum levels of granzyme A and H, as well as CSF1, carbonic anhydrase IX and TNF‐like weak inducer of apoptosis (TWEAK) [169]. The exact mechanism of these findings remains to be elucidated. Overexpression of T‐bet or Eomes significantly enhances NK cell function, including increased IFN‐γ production and cytotoxicity. In particular, Eomes‐overexpressing NK cells exhibit enhanced antibody‐dependent cellular cytotoxicity [170]. Moreover, all type 1 innate lymphoid cells (ILC1) express the transcription factor T‐bet, whereas pNK cells express Eomes. Deletion of the Eomes allele using NKp46‐Cre cells at the onset of type 1 ILC maturation severely impairs the development of pNK cells [171].

#### 3.1.2 dNK Cells

NK cells are the most abundant immune cells in the decidua and are traditionally classified into three subtypes: dNK1, dNK2, dNK3, and dNK4 [172, 173]. Abnormal distribution of these subtypes significantly affects pregnancy outcomes. In patients with RSA, dNK1 cells are markedly reduced, whereas dNK2 cells show a slight increase, and dNK3 cells are significantly elevated [173, 174]. dNK2 and dNK3 cells overexpress the chemokines XCL1 and CCL5 [80], which may recruit a large number of cytotoxic CD56^dim^ CD16^+^ cells [172, 175, 176, 177]. However, the correlation between these findings remains controversial [178]. The dNK4 subset expresses FCGR3A/CD16 and is specific to the decidual tissue of RSA patients. It accumulates more in the decidual compact layer microenvironment near blood vessels, with higher expression of pro‐inflammatory factors CXCL8 and IFIT3, indicating cytotoxic activity [172]. Recent research has identified a significant increase in IL‐22‐producing NK22 cells [179, 180], and CD18^+^ dNK cells [181] in the decidua of patients with RSA; however, their pathogenic mechanisms require further investigation.

During early pregnancy, decidual cells establish a series of microcellular contacts to create a favourable endometrial microenvironment. In miscarriage samples, this cellular communication appears disrupted and is closely associated with an increase in the number of NK cells. Such changes may disturb the normal process of embryo implantation, adversely affect embryo development and result in miscarriage [182]. Mechanistically, one factor contributing to these outcomes is the imbalance in the expression of surface receptors on dNK cells. For instance, the abnormal expression of the activating receptor KIR2DS1, when interacting with the ligand HLA‐C2, causes adverse pregnancy outcomes [183, 184, 185].

NKp46, a natural cytotoxic receptor, is categorised into NKp46^dim^ and NKp46^bright^ NK cells. NKp46^dim^ NK cells are significantly increased in patients with reproductive failure, producing IFN‐γ and TNF‐α and contributing to cytotoxicity. Conversely, NKp46^bright^ NK cells are significantly reduced in patients with reproductive failure, resulting in insufficient TGF‐β secretion [10, 186]. TNF‐α, IL‐1β and LPS stimulation can upregulate the expression of the aryl hydrocarbon receptor (AhR) in dNK cells in vitro, enhancing NK cell cytotoxicity and inducing RSA [187]. Reducing uNK cell counts, neutralising TNF‐α or administering IL‐10 can improve pregnancy outcomes [188, 189].

TGF‐β acts on decidual cells by activating activin receptor‐like kinase 5, inducing the expression of growth factors and cytokines, promoting epithelial proliferation and trophoblast development during pregnancy. Insufficient expression of TGF‐β causes the disorganisation of trophoblast cells and impaired spiral artery remodelling [190]. Additionally, dNK cells exhibit abnormal cytokine expression. The level of endogenous cannabinoid anandamide (AEA) is higher in the decidua of patients with RSA, which induces dNK cells to secrete TNF‐α, interfering with the viability and differentiation of ESCs and resulting in luteal dysfunction [191, 192].

Villus‐derived exosomes (vEXOs) contain various molecules that can be effectively internalised by dNK cells. Under normal conditions, the internalised miR‐29a‐3p binds to the 3′ untranslated region (UTR) of IFN‐γ mRNA to inhibit its production. However, in patients with RSA, this process is hindered, resulting in significantly elevated IFN‐γ levels [9]. IFN‐γ, as a negative regulator of invasion, inhibits trophoblast cell invasion in a dose‐dependent manner [193]. By regulating CX3CL1 expression in ESCs, IFN‐γ promotes the recruitment of CD49b^+^ dNK cells, induces high expression of their Ly‐49 receptors and strongly enhances the activation of complement component C3 and the enzyme adipsin in the miscarriage placenta, resulting in foetal loss [194, 195]. Granulysin (GNLY) produced by NK cells attacks trophoblast cells, leading to extravillous trophoblast (EVT) apoptosis [196]. However, insufficient expression of IFN‐γ and granzyme B by CD56^+^ dNK cells reduces the invasive and migratory ability of trophoblast cells, causing RSA [197].

The expression level of peroxiredoxin‐2 in uNK cells is significantly decreased, increasing their cytotoxicity and leading to miscarriage [198]. Pigment epithelium‐derived factor (PEDF), a multifunctional glycoprotein derived from dNK cells, plays a broad role in angiogenesis, inflammation, metabolic homeostasis and immune regulation. PEDF maintains the homeostasis of DSCs and immune balance at the maternal–fetal interface during early pregnancy. In patients with RSA, PEDF expression is significantly reduced, weakening the anti‐inflammatory and anti‐apoptotic effects mediated by dNK cells [199]. The decrease in PBX homeobox 1 (PBX1) expression in dNK cells leads to insufficient activation of AKT1, which results in inadequate expression of pleiotrophin and osteoglycin, thereby contributing to RSA [200]. Furthermore, the secretion of inhibitory factors by decidual cells is reduced, and decidualisation defects are widely considered an important cause of spontaneous abortion. Autophagy of DSCs is enhanced by activating the melanocyte‐inducing transcription factor (MITF)‐herpes virus entry mediator (TNFRSF14/HVEM) signalling pathway and upregulating MMP9, promoting the adhesion of DSCs to NK cells during normal pregnancy. However, in patients with RSA, autophagic activity of DSCs is significantly reduced, with decreased expression of MITF, TNFRSF14/HVEM and MMP9, resulting in insufficient NK cell retention and poor pregnancy outcomes [201]. After autophagy inhibition in DSCs, the expression of IGF‐2 increases, enhancing NK cell cytotoxicity, including the release of NKG2D, NKp46, CD107 and perforin, which mediate trophoblast apoptosis [202, 203]. Simultaneously, the CXCR4^+^ dNK cell phenotype undergoes an abnormal shift, with decreased granzyme B expression, potentially resulting in miscarriage [204, 205]. Reduced HLA‐G expression in exfoliated trophoblast cells causes maternal immune dysregulation [206], as it fails to suppress IL‐2‐mediated NK cell cytotoxicity [207, 208]. Decreased integrin αvβ3 disrupts decidual tissue homeostasis, leading to histological disarray and impaired cell proliferation, ultimately increasing pathogenic NK cells and serum IFN‐γ levels [209].

Reduced hyaluronan synthesis affects implantation by inducing vascular permeability, defective vascular sinus fold formation and impaired implantation [210]. Lower EVT B7‐H3 content inhibits the RhoA/ROCK2 signalling pathway, reducing trophoblast migration and invasion while increasing dNK secretion of IL‐8 and IP‐10 [211]. Studies show that reduced brain and muscle AhR nuclear translocator‐like protein 1 expression in the endometrium inhibits uNK function, impairing placental vascular formation and pregnancy maintenance [212]. Ubiquitin C‐terminal hydrolase L1, a deubiquitinating enzyme, is significantly downregulated in RSA meconium. Its blockade inhibits the JAK2/STAT3 pathway, reducing cytokines that regulate dNKs (CXCL12, IL‐15, TGF‐β) and leading to poor outcomes [213].

Inadequate dNK recruitment and reduced stromal IGF‐1 expression impair uterine metamorphosis [214, 215]. Additionally, dramatic downregulation of PR and progesterone‐induced blocking factor (PIBF) increases CD56^+^ NK cells, reduces protective anti‐inflammatory cytokines (IL‐10, IL‐15), and upregulates pro‐inflammatory cytokines (TNF‐α, IFN‐γ, NF‐κB) [216, 217]. Neuropilin‐1 (NRP1) is abnormally elevated in metaphase immune cells in association with miscarriage; however, its mechanism remains unclear [218].

### 3.2 RIF and NK Cells

#### 3.2.1 pNK Cells

Patients with RIF exhibit a pNK cell profile similar to that of patients with RSA. Women with RIF demonstrate increased concentrations of activated CD56^dim^ CD69^+^ NK cells in the peripheral blood [219, 220, 221]. However, some studies did not find this difference [222, 223]. Elevated levels of CD8^−^ NK cells in peripheral blood have been observed, which can lyse target cells and are associated with high levels of effector apoptosis [224]. The elevation of these pNK cells promotes the expression of TNF‐α, IFN‐γ, granzyme B and GM‐CSF or inhibits the expression of membranous Tim‐3, IL‐4 and IL‐10, which can negatively impact ovarian function and reproduction [225, 226, 227, 228, 229, 230].

#### 3.2.2 dNK Cells

**FIGURE 3:**
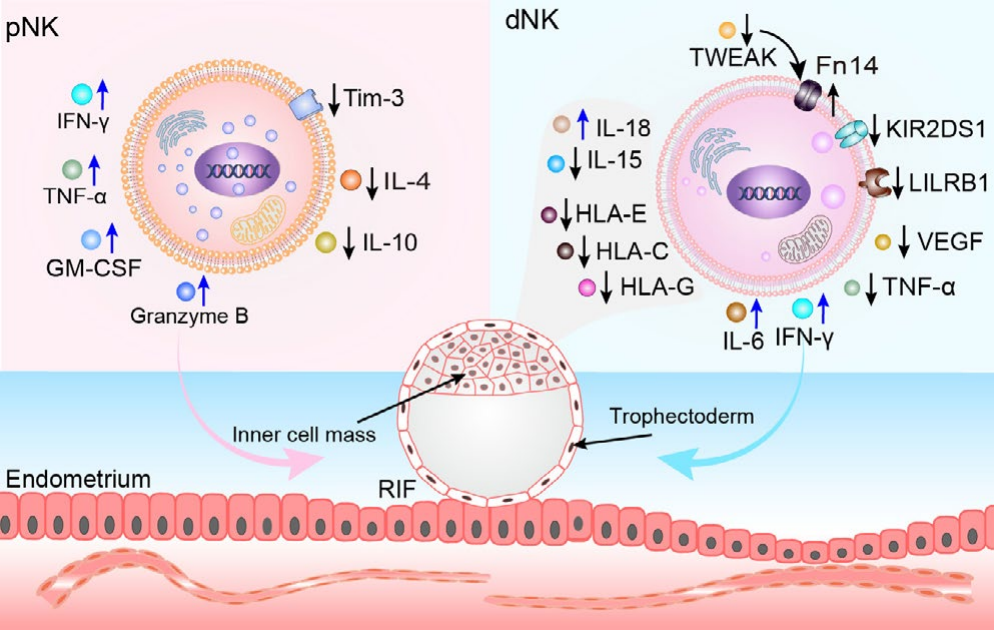
Role of NK cells in RIF. In RIF, the concentration of CD56^dim^ CD69^+^ NK cells and CD8^−^ NK cells increases. The elevation of these pNK cells promotes the expression of TNF‐α, IFN‐γ, granzyme B and GM‐CSF, or inhibits the expression of membranous Tim‐3, IL‐4 and IL‐10, which can negatively impact ovarian function and reproduction. In patients with RIF, the significant increase in CD56^dim^ dNK cells in the uterus leads to their hyperactivity, reducing endometrial tolerance and ultimately resulting in RIF. The decreased expression of inhibitory receptors on uNK cells, such as KIR2DL1/S1 and LILRB1, along with insufficient expression of HLA‐C, HLA‐G and HLA‐F in trophoblast cells, reduces uNK cell degranulation activity and decreases VEGF and TNF‐α production, contributing to RIF. Additionally, an increased number of CD16^+^ uNK cells and a decreased proportion of CD56^bright^ uNK cells lead to elevated IL‐6 levels. Moreover, decreased IL‐15 and increased IL‐18 expression in trophoblast cells result in insufficient proliferation and activation of uNK cells, while IL‐18, in combination with IL‐2, enhances IFN‐γ production and the cytotoxic activity of uNK cells. Furthermore, the decreased expression of TWEAK in uNK cells and the increased expression of its receptor Fn14 alter uNK cytotoxicity, disrupt decidual homeostasis and ultimately lead to foetal rejection.

Compared to controls, uterine CD56^dim^ NK cells are significantly higher in patients with RIF, and their overactivity leads to reduced endometrial tolerance, contributing to RIF (Figure 3) [3, 231, 232]. Inhibitory receptors on uNK cells, such as KIR2DL1/S1 and LILRB1, are decreased, along with reduced expression of HLA‐C, HLA‐G and HLA‐F. This under‐expression results in significantly reduced uNK degranulation activity, diminished VEGF and TNF‐α production, and ultimately RIF [233, 234, 235, 236]. Furthermore, increased counts of CD16^+^ uNK cells and a decreased CD56^bright^ uNK fraction lead to elevated IL‐6 levels [237]. Reduced secretion of CXCL12 and IL‐15 by endometrial epithelial cells impairs the recruitment of CD49a^+^ CXCR4^+^ NK cells [238]. Trophoblast cells exhibit decreased expression of IL‐15 and increased IL‐18. The former results in insufficient proliferation and activation of uNK cells, while the latter, in combination with IL‐2, enhances uNK IFN‐γ production and cytotoxic activity [239, 240, 241].

TWEAK and its receptor fibroblast growth factor‐inducible molecule 14 (Fn14) are also implicated [242]. TWEAK provides protection against the deleterious effects of TNF‐α during implantation [243], but the decreased expression of TWEAK in uNK cells and the increased expression of its receptor Fn14 alter the cytotoxicity of uNK cells, disrupting decidual homeostasis and ultimately leading to foetal rejection [244]. Conversely, overactivation of TWEAK is strongly associated with stromal cell death and pregnancy failure [245].

The endoplasmic reticulum (ER) aminopeptidases ERAP1 and ERAP2 modify polypeptides to generate stable antigenic epitopes displayed to T‐cell receptors via HLA class I molecules [246]. The ERAP1 genotype, TT rs30187, is associated with an increased risk for RIF in women with the male HLA‐C1C2 genotype [247]. Additionally, hormonal stimulation with gonadotropins alters the abundance of maternal immune cells, including uNK cells, and reduces their ability to promote EVT invasion [248].

## 4 Pathogenic Factors

**FIGURE 4:**
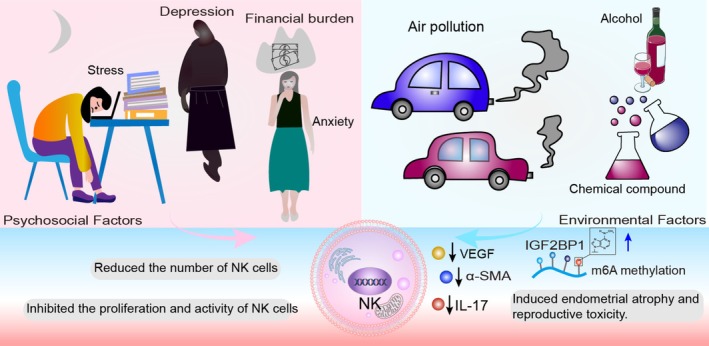
Psychosocial and environmental factors associated with pregnancy failure. Psychosocial factors, including workplace competition, high treatment costs, depression and anxiety, reduce NK cell numbers and inhibit their proliferation and activity. Environmental factors, such as air pollution, alcohol consumption and exposure to synthetic chemicals, suppress IL‐17 signalling in NK cells, decreasing α‐SMA and VEGF levels. Abnormal m6A methylation accumulation at the IGF2BP1 promoter induces endometrial atrophy, enhances NK cell cytotoxicity and contributes to pregnancy failure.

RSA and RIF involve complex mechanisms influenced by multiple factors. Psychological and social factors, such as stress and emotional fluctuations, affect pregnancy through the neuroendocrine–immune axis. Environmental factors, including pollution, smoking and occupational exposure, can disrupt embryo implantation and development. Autoimmune disorders, such as antiphospholipid syndrome (APS) and thyroid autoimmunity (e.g., anti‐TPO antibodies), may cause uterine environment changes (Figure 4). Additionally, microbial infections, obesity, gestational diabetes, uterine anatomical abnormalities (e.g., intrauterine adhesions) and hypercoagulable states increase the risk of embryo loss. These factors are intricately interrelated and require precise assessment and targeted treatment based on specific underlying causes.

### 4.1 Psychosocial Factors

The psychosocial effects of RSA and RIF are complex and profound. High expectations of childbearing from couples and families may impose significant psychological stress on patients, who may be perceived as “incapable” or “unlucky,” resulting in a loss of self‐esteem [249]. In the workplace, the dual challenges of career competition and fertility issues exacerbate the psychological burden [250]. Furthermore, the high cost of diagnosing and treating RSA and RIF, including procedures such as in vitro fertilisation (IVF), genetic screening and immunotherapy, places significant financial strain on families with limited resources, increasing anxiety and depression risks [251].

Psychological stress, along with personality traits such as neuroticism, depressive symptoms and low self‐esteem, disrupts immune system function and impairs endometrial receptivity by suppressing NK cell proliferation and activity [252]. Patients with infertility experience significant anxiety, while couples experiencing treatment failure are at higher risk of depression. Excessive psychological stress is a critical factor contributing to recurrent miscarriages. Stress‐induced overproduction of serum cortisol reduces pNK cell numbers in patients with RSA through glucocorticoid (GC) receptor pathways [253]. However, moderate psychological stress may improve pregnancy outcomes by increasing pNK cell numbers; however, this association remains controversial.

Psychiatric interventions alleviate psychological distress, such as anxiety and depression, in patients with RSA while reducing NK cell activity [254]. RSA and RIF are not merely physiological issues; they profoundly impact the psychological and social lives of patients. Collective efforts from society, families, healthcare institutions and patients are essential to create an environment of understanding and support. Such efforts can mitigate the negative effects of psychosocial factors, improve patients' quality of life and enhance pregnancy success rates.

### 4.2 Environmental Factors

Early pregnancy is considered the period when the foetal immune system is most sensitive to air pollutants. Maternal exposure to air pollution can disrupt immune cell function and increase the risk of RSA. Severe air pollution may exert both acute and chronic effects on the immune systems of pregnant women and foetuses. For instance, elevated levels of NK cells in umbilical cord blood have been linked to air pollution exposure [255]. Conversely, other studies have demonstrated a significant association between higher exposure to traffic‐related pollutants (e.g., nitrogen dioxide concentrations ≥ 36.4 μg/m^3^) during foetal development and decreased NK cell levels in umbilical cord blood, indicating a complex mechanism [256, 257].

Single‐cell RNA sequencing (scRNA‐seq) studies have shown that fine particulate matter (PM2.5) reduces uNK cell numbers and inhibits IL‐17 signalling pathways, potentially inducing endometrial atrophy and reproductive toxicity [258]. Additionally, comparative analyses of decidual tissues from patients with induced abortion and RSA using RNA sequencing, reduced representation bisulfite sequencing and scRNA‐seq revealed a significant positive correlation between maternal exposure to air pollutants in the year preceding pregnancy and during early pregnancy with RSA risk. This effect is likely mediated by altered methylation levels of the IGF2BP1 promoter, resulting in pregnancy loss [259].

Alcohol exposure also significantly affects maternal uterine tissues. Female mice consuming 10% ethanol exhibited reduced vascular lumen dilation at implantation sites, decreased α‐smooth muscle actin (α‐SMA)‐positive spiral arteries and diminished uNK cell numbers in the decidua compared to controls. Reduced expression of VEGF and its receptor KDR (VEGFR2) in decidual tissues further impairs angiogenesis, providing a mechanistic basis for defective decidual vascular development in alcohol‐exposed pregnancies [260].

Moreover, exposure to a mixture of chemicals throughout life may adversely affect pregnancy outcomes. Certain endocrine‐disrupting chemicals, such as benzophenone‐3 and bisphenol A (BPA), can harm foetal and placental development by increasing uNK cell numbers [261]. High BPA exposure levels are also associated with the production of antinuclear antibodies, which may contribute to RSA [262].

### 4.3 Autoimmune Diseases

APS is an autoimmune disorder that can cause severe, life‐threatening complications during pregnancy. Antiphospholipid antibodies (aPLs) have been detected in patients with RSA and IVF failure. Anti‐phosphatidylethanolamine and anti‐phosphatidylserine are directly associated with increased pNK cell activity [263], a finding corroborated by other studies [264].

In patients with APS, the number of CD3^−^ CD16^+^ CD56^dim^ NK cells in peripheral blood is significantly elevated. Moreover, the expression of the inhibitory receptor NKG2A is markedly decreased, whereas the activating receptor NKG2D is upregulated [265]. Women with anti‐SSA/SSB antibodies also exhibit higher levels of peripheral blood cytokines, including TNF‐α and IL‐17A [266]. aPLs stimulate NK cell degranulation, inducing antibody‐dependent cellular cytotoxicity. This process is characterised by increased degranulation and elevated expression of CD11b, CD69 and NKG2D [267].

Interestingly, menstrual blood, which can be collected noninvasively, serves as a unique window into the immune state of the endometrium. It contains a significant number of CD49a^+^ NK cells. Compared to traditional CD49a^−^ NK cells, CD49a^+^ NK cells exhibit an exhausted phenotype with higher expression levels of immune checkpoint molecules such as programmed cell death protein 1 and Tim‐3. These cells are also associated with increased secretion of IFN‐γ, TNF‐α and granzyme B [268].

### 4.4 Thyroid Autoimmunity

The relationship between thyroid autoimmunity, RSA and poor pregnancy outcomes, including RIF, has received considerable attention. Thyroid autoimmunity primarily refers to the presence of thyroid autoantibodies, such as anti‐thyroid peroxidase and anti‐thyroglobulin antibodies, commonly observed in thyroid disorders like Hashimoto's thyroiditis. Patients with thyroid autoimmunity often exhibit immune system abnormalities, which may disrupt immune tolerance in the placental microenvironment, thereby increasing the risk of RSA and RIF [269].

In autoimmune thyroid diseases such as Hashimoto's thyroiditis and Graves' disease, pNK cell activity is increased and pro‐inflammatory cytokines such as IL‐6 and TNF‐α are elevated, potentially impairing the embryonic development environment [270]. However, this hypothesis remains controversial. Although pNK cells in patients with Hashimoto's thyroiditis show a trend of increased cytotoxicity towards foetal thyroid cells, this effect is not statistically significant [271].

Non‐autoimmune thyroid disorders have also been associated with increased risks of infertility and RSA. Subclinical hypothyroidism (SCH) is the most common thyroid disorder in women with RSA. Compared to patients with thyroid autoimmunity, women with SCH and RSA exhibit higher NK cell levels. This suggests a link between NK cell levels and thyroid function, observable in both autoimmune and non‐autoimmune thyroid disorders [272].

### 4.5 Microbiota

**FIGURE 5:**
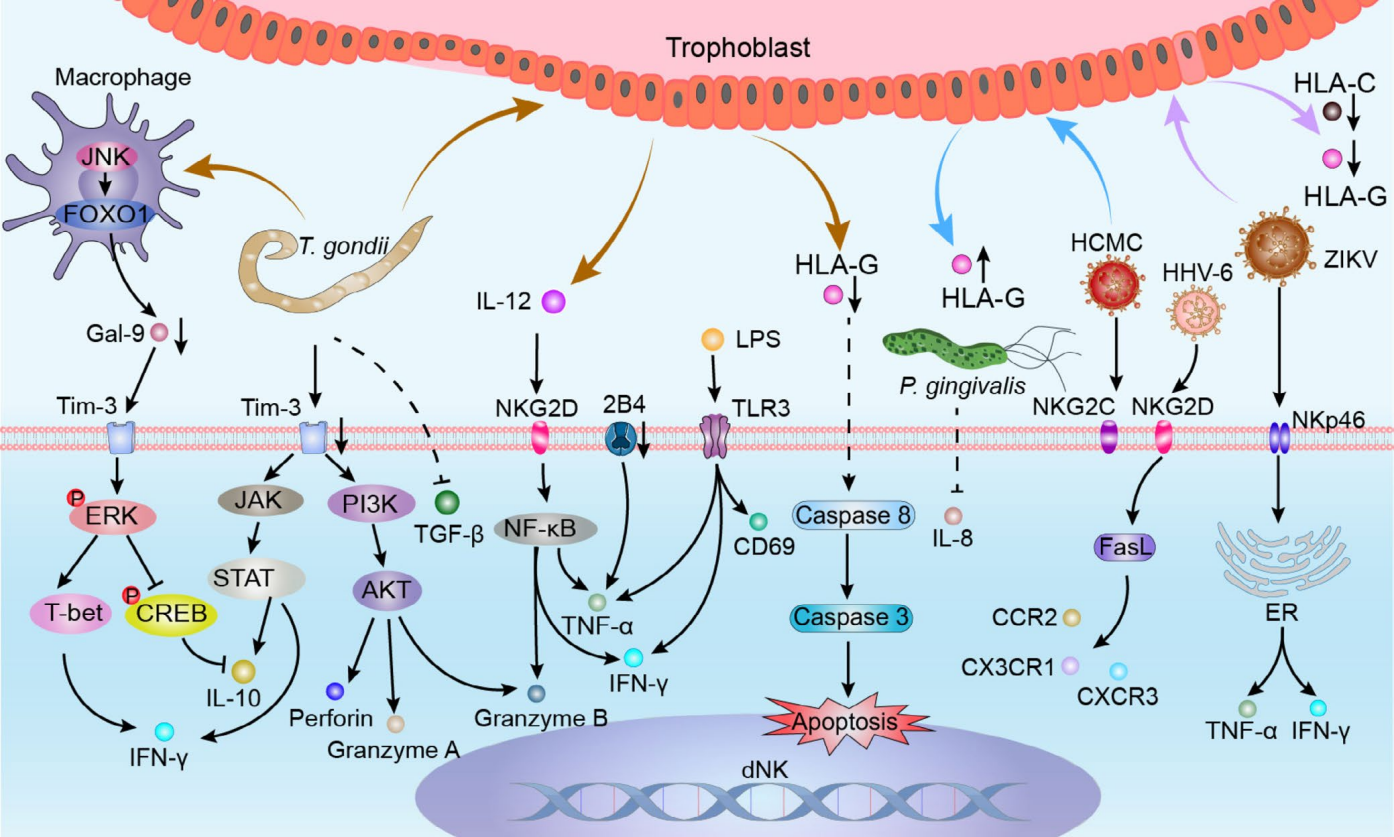
Role of microorganisms in pregnancy failure. *Toxoplasma gondii* infection stimulates decidual dendritic cells (dDCs) to secrete IL‐12, which enhances NKG2D expression in dNK cells and activates the NF‐κB signalling pathway. This results in increased secretion of IFN‐γ, TNF‐α and granzyme B, heightening dNK cell cytotoxicity. TGF‐β signalling is suppressed during infection, weakening maternal‐foetal immune tolerance. *T. gondii* infection reduces Tim‐3 expression while promoting NKG2D expression in dNK cells, activating the PI3K‐AKT and JAK‐STAT pathways. These changes upregulate granzyme B, granzyme A, perforin, IFN‐γ and IL‐10 production, contributing to adverse pregnancy outcomes. Additionally, *T. gondii* activates the JNK/FOXO1 pathway, reducing Gal‐9 expression in decidual macrophages. The loss of Gal‐9 disrupts the Gal‐9/Tim‐3 interaction, impairing dNK cell function by inducing ERK phosphorylation, inhibiting p‐CREB and IL‐10 expression and promoting T‐bet and IFN‐γ expression, further exacerbating adverse outcomes. The infection downregulates 2B4 expression, increasing TNF‐α and IFN‐γ secretion. Furthermore, *T. gondii* elevates HLA‐G levels in trophoblast cells, increases caspase‐3 and caspase‐8 activity in dNK cells, and induces apoptosis. *Porphyromonas gingivalis* suppresses IL‐18 production by dNK cells, impairing spiral artery remodelling and reducing EVT invasion. Lipopolysaccharide (LPS) activates TLR3 in dNK cells, upregulating CD69, TNF‐α and IFN‐γ expression. Human cytomegalovirus (HCMV) enhances NK cell cytotoxicity via NKG2D and NKG2C, while HHV‐6 increases chemokine receptor expression (CCR2, CXCR3 and CX3CR1) through the NKG2D‐FasL pathway. Zika virus (ZIKV) induces endoplasmic reticulum (ER) stress, downregulating inhibitory receptor ligands HLA‐C and HLA‐G on trophoblast cells, rendering them targets for dNK cells. ER stress activates NKp46, leading to high levels of IFN‐γ and TNF‐α production by dNK cells, which ultimately kill ZIKV‐infected trophoblasts.

An imbalance in the microbiota may affect the immune environment of the endometrium, thereby increasing the risk of RSA and RIF. Overgrowth of specific pathogens and harmful microorganisms in the uterus can cause local inflammatory responses and disrupt embryo implantation and development. Restoring uterine microbiota balance may help improve clinical outcomes of RSA and RIF (Figure 5).

*Toxoplasma gondii*, an intracellular parasite, can cause adverse pregnancy outcomes through vertical transmission [273]. *T. gondii* infection leads to hyperactivation of dNK cells. The infection stimulates DCs to secrete IL‐12, increasing the expression of the dNK activating receptor NKG2D, which promotes the secretion of IFN‐γ, TNF‐α and granzyme B, thereby enhancing dNK cell cytotoxicity [274, 275]. TGF‐β1 treatment can mitigate *T. gondii*‐induced abnormal pregnancy outcomes by reducing NKG2D/DAP10 signalling and the cytotoxicity of dNK killer subsets [276]. *T. gondii* infection also reduces Tim‐3 expression on dNK cells, significantly activating the PI3K‐AKT and JAK–STAT signalling pathways, which upregulate the production of granzyme B, granzyme A, perforin, IFN‐γ and IL‐10, ultimately leading to abnormal pregnancy outcomes [277].

Furthermore, the infection activates the JNK/FOXO1 signalling pathway to suppress Gal‐9 expression in decidual macrophages. Reduced Gal‐9 levels disrupt Gal‐9/Tim‐3 interactions, resulting in dNK cell dysfunction. This dysfunction is characterised by increased ERK phosphorylation, suppression of phosphorylated CREB (p‐CREB), and IL‐10 expression and enhanced T‐bet and IFN‐γ expression, which contribute to poor pregnancy outcomes [278]. Additionally, studies reveal significant downregulation of 2B4 after *T. gondii* infection. In 2B4‐deficient pregnant mice, TNF‐α and IFN‐γ expression and dNK cytotoxicity are increased [279].

On the other hand, *T. gondii* infection increases HLA‐G levels in trophoblast cells, mediating the elevation of apoptotic proteins caspase‐3 and caspase‐8 in dNK cells, inducing apoptosis and exacerbating adverse pregnancy outcomes [280]. Uterine epithelial IFN‐ε enhances uNK cell accumulation by increasing IL‐15 expression in local immune cells, promoting IFN‐γ production and cytolytic activity [281]. While this activity is crucial for preventing systemic *Chlamydia muridarum* transmission, it may cause adverse pregnancy outcomes [282].

In patients with RSA, the abundance of *Lactobacillus*, *Curvibacter*, *Gardnerella vaginalis* and other gram‐negative anaerobes is significantly increased, closely associated with elevated pNK cell numbers [283, 284]. Intrauterine infection with *Porphyromonas gingivalis* reduces IL‐18 expression in dNK cells, impairs spiral artery remodelling, increases smooth muscle cell retention in spiral arteries, and decreases EVT invasion [285]. Bacterial and viral infections activate host innate immune responses via Toll‐like receptor (TLR)‐mediated signalling pathways, contributing to pregnancy failure [286].

Lipopolysaccharide (LPS), a major gram‐negative bacterial outer membrane component, acts as a potent immune stimulant by interacting with TLRs and inducing strong innate immune responses. TLR3 activation upregulates CD69 on dNK cells and significantly increases intracellular TNF‐α and IFN‐γ levels [287, 288]. LPS may also induce elevated intrauterine IL‐15 expression, causing dysregulated dNK activity, increased cytotoxicity towards placental or embryonic tissues, and subsequent miscarriage [289]. Pregnancy‐trained dNK cells bind to the Fap2 protein of *Fusobacterium nucleatum* via surface glycan Gal‐GalNAc, increasing GNLY production to protect the foetus from *F. nucleatum* infection [290]. dNK cells can also selectively transfer GNLY to trophoblast cells via nanotubes, killing intracellular *Listeria monocytogenes* , significantly reducing placental and foetal infection loads, and improving pregnancy success rates. This specific GNLY transfer mechanism also applies to pNK cells, enhancing systemic antibacterial immunity [291].

Human cytomegalovirus (HCMV) is a common cause of intrauterine viral infections, severely affecting ovarian cells except follicular cells. HCMV selectively and intensely infects luteal cells, leading to progesterone deficiency and miscarriage [292]. When dNK cells encounter HCMV‐infected decidual fibroblasts, they transform into cytotoxic effector cells. Activating receptors, including NKG2D and CD94‐NKG2C/2E, are involved in this cytotoxic transformation [293]. During early pregnancy, HCMV‐infected EVT cells continue to express HLA‐G and CEACAM1 molecules, inhibiting maternal NK cell lysis, proliferation and cytokine secretion [294, 295]. HCMV also suppresses EVT proliferation and invasion, disrupting maternal‐foetal immune cross‐talk [296].

ILC‐derived NK cells, transcriptionally similar to CD56^bright^ NK cells, produce large amounts of IFN‐γ during *Salmonella* and herpes simplex virus infections [297]. Pathways in uNK cells enhance cytotoxicity and increase CCR2, CXCR3 and CX3CR1 chemokine receptor expression [298].

Zika virus (ZIKV) infects foetal trophoblast cells, causing placental damage and birth defects. ZIKV infection induces ER stress in trophoblast cells, downregulating HLA‐C/G receptor ligands and making trophoblast cells targets for dNK cells. ER stress activates the dNK activating receptor NKp46, elevating IFN‐γ and TNF‐α levels and triggering dNK‐mediated killing of ZIKV‐infected trophoblast cells [299, 300, 301]. While research on ZIKV's correlation with RSA or RIF is limited, it may contribute to adverse pregnancy outcomes.

Severe acute respiratory syndrome coronavirus 2 (SARS‐CoV‐2), the cause of COVID‐19, poses significant public health concerns. The immunological changes associated with pregnancy and SARS‐CoV‐2 infection remain incompletely understood. COVID‐19 infection during the first trimester does not significantly alter leukocyte or cytokine levels at the maternal‐foetal interface [302, 303]. Maternal IgA/IgM neutralising antibodies and low ACE2 expression in trophoblast cells provide some placental protection [304, 305]. However, symptomatic infected women show elevated IFN‐γ and TNF‐α levels in NK cells, increasing foetal death risk. Newborns of SARS‐CoV‐2‐infected mothers exhibit fewer, less functional NK cells [306]. COVID‐19 vaccination has been shown to improve pregnancy outcomes, with vaccinated frozen embryo transfer patients having higher clinical and live birth rates than unvaccinated patients [307].

### 4.6 Obesity

Over one‐fifth of women of reproductive age in North America are obese, increasing their risk of various chronic diseases. Obesity disrupts immune regulation and causes NK cell dysfunction, potentially heightening the risk of RSA and RIF. Obesity significantly reduces the number of uNK cells and impairs uterine artery remodelling. Mechanistically, obesity increases platelet‐derived growth factor (PDGF) receptor expression in uNK cells, leading to an exaggerated response to PDGF and promoting excessive decorin (DCN) expression. DCN strongly inhibits placental development by limiting trophoblast cell survival [308].

Diet‐induced obesity increases progesterone secretion and alters the response of uNK cells. Compared to control mice, high‐fat diet‐induced obesity significantly reduces the proportion of uNK cells and decreases IFN‐γ expression, hindering uterine spiral artery remodelling [309]. In obese mothers, uNK cell activation through KIR2DS1 enhances HLA‐C2 activation and promotes TNF‐α production [310]. In the normal pregnancy group, high‐fat diet‐induced obesity upregulates NK cell activation receptor NKp46. However, in the miscarriage‐prone group, obese mice exhibit more immature NK cells with reduced activity [311].

Compared to the normal‐weight group, obese patients with RSA show significantly increased peripheral Th cells, while cytotoxic T cells and NK cells are decreased. In patients with RIF, the proportion of uNK cells, M2 macrophages and Tregs significantly decreases as BMI increases. However, in patients with RSA, both uNK cells and M2 macrophages are reduced [312]. Other studies suggest that RIF is not associated with uNK cells but is instead linked to an increased density of immune‐suppressive Tregs and M2 macrophages in the endometrium [313, 314].

**FIGURE 6:**
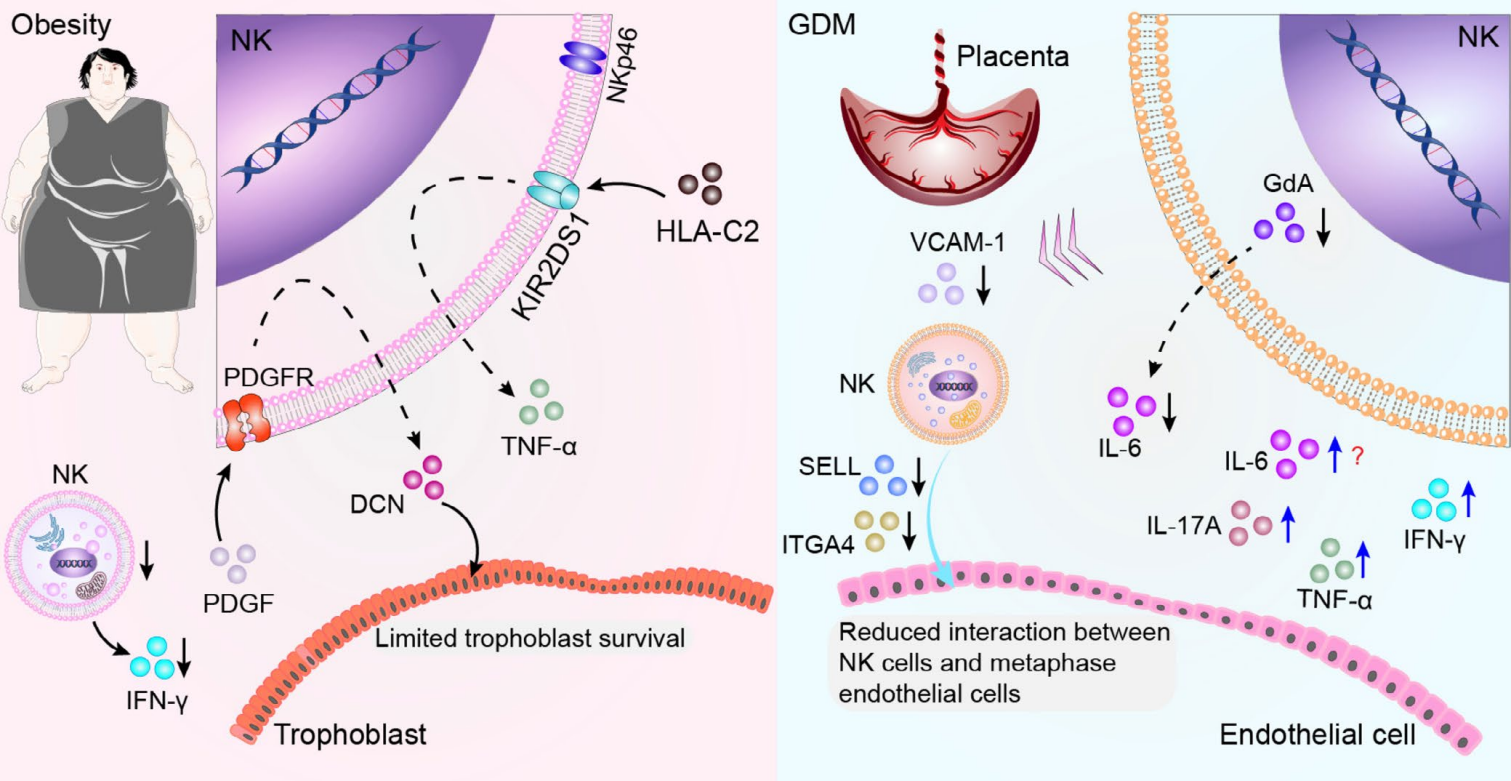
Role of NK cells in pregnancy failure in women with obesity and GDM. High‐fat diet‐induced obesity significantly reduces the proportion of uNK cells and lowers IFN‐γ expression, impairing uterine spiral artery remodelling. Platelet‐derived growth factor (PDGF) activates PDGF receptor (PDGFR), driving excessive decorin (DCN) expression and restricting trophoblast survival. Interaction between HLA‐C2 and KIR2DS1 on uNK cells promotes TNF‐α production, while obesity upregulates NKp46, enhancing NK cell activation. In women with GDM, pNK cell numbers decrease, and reduced levels of GdA impair IL‐6 secretion, contributing to foetal growth restriction and RSA. Elevated IL‐6 and IL‐17A levels have also been reported in some studies. Insufficient expression of vascular cell adhesion molecule‐1 (VCAM‐1) in the decidua impairs uNK cell recruitment. The recruited uNK cells are smaller, contain fewer cytoplasmic granules and exhibit diminished functionality. Additionally, disrupted interactions between L‐selectin (SELL) and integrin alpha 4 (ITGA4) reduce adhesion between NK cells and decidual endothelial cells, further compromising their role in pregnancy maintenance.

Lactoferrin has restored normal blood glucose levels and enhanced ovarian function in high‐fat diet‐induced obese mice [315]. However, there is no current evidence linking lactoferrin to improved NK cell function (Figure 6).

### 4.7 Gestational Diabetes Mellitus

Gestational diabetes mellitus (GDM) is characterised by hyperglycemia during pregnancy, leading to dysfunction in various cells, particularly immune cells. In GDM mice, the proportion of cytotoxic CD27‐CD11b^+^ dNK cells significantly increases, while the proportion of regulatory CD27‐CD11b^−^ dNK cells decreases. Similarly, peripheral blood NK cell subsets in patients with GDM exhibit the same trend [316]. GdA, a glycoprotein abundant in the decidua with glycosylation‐dependent immunomodulatory activity, shows reduced levels of α2‐6‐sialylated and high‐mannose glycans in diabetic pregnancies. This alteration weakens pNK cells' ability to secrete IL‐6, with insufficient IL‐6 associated with foetal growth restriction and RSA [317]. GDM is typically accompanied by obesity. In obese women with GDM, the proportions of CD56^dim^CD16^+^ NK cells in peripheral blood and placental NK cells are significantly reduced, whereas IL‐6 and IL‐17A levels are elevated [318]. Depletion of NK cells in GDM increases the risk of embryo loss [319]. However, the absolute number and proportion of NK lymphocytes (CD57) are significantly higher in patients with GDM compared to normal pregnancies [320].

In type 1 diabetes mellitus (T1DM) models, uNK cell numbers increase, but stromal trophoblast invasion and spiral artery remodelling are severely impaired [321]. T1DM mice exhibit abnormal expression of MAdCAM‐1 and reduced vascular cell adhesion molecule 1 (VCAM‐1) in the decidua, leading to insufficient uNK cell recruitment. These uNK cells are smaller, contain fewer cytoplasmic granules, and exhibit diminished spiral artery remodelling capacity. Additionally, significantly elevated IFN‐γ levels are observed in the decidual basalis, though the underlying mechanisms remain unexplored [322]. T1DM also disrupts the interaction between CD56^+^ NK cells in the blood and decidual endothelial cells by impairing homing receptor interactions involving L‐selectin (SELL) and integrin alpha 4 (ITGA4) [323]. In patients with type 2 diabetes mellitus, higher proportions of CD16^+^CD56^−^ and CD16^−^CD56^+^ cells are found in cord blood. The outer placenta layer shows an increase in CD16^+^CD56^−^ cells. Hyperglycemia creates an inflammatory environment in the placenta, leading to increased secretion of TNF‐α, IL‐17 and IFN‐γ [324].

### 4.8 Endometrial Abnormalities

Endometrial abnormalities, including chronic endometritis (CE), endometriosis (EMs) and uterine injury, are critical factors contributing to RSA and RIF. CE, a low‐grade inflammatory condition caused by infectious pathogens, is characterised by an abundance of plasma cells in the endometrial glands and stroma. This leads to an abnormal endometrial environment, immune dysregulation and impaired NK and Treg cell function, thereby increasing the risk of RSA and RIF. EMs disrupt endometrial receptivity and embryo implantation through local inflammation and immune disturbances, involving abnormal proportions of NK cells and macrophages, imbalanced cytokine production (e.g., elevated IL‐6 and TNF‐α), and impaired angiogenesis and tissue remodelling. Uterine injury, often caused by repeated curettage, surgeries or infections, can result in endometrial fibrosis, inflammation and reduced blood flow. These changes further impair embryo implantation and development, alter the local immune environment and cause uNK cell dysfunction. Collectively, these abnormalities increase the risk of RSA and RIF by altering the uterine structure and immune conditions. Treatments targeting these mechanisms, including anti‐inflammatory therapy, immune modulators and endometrial repair techniques, may improve pregnancy outcomes.

CE is a chronic inflammatory condition of the endometrium, typically caused by persistent low‐level pathogenic infections. In recent years, the association between CE and adverse reproductive outcomes, such as RSA and RIF, has gained significant attention. Studies have shown that patients with CE exhibit significantly higher levels of immune cell infiltration, including uNK cells, in the endometrium compared to individuals without CE. This excessive immune cell infiltration reduces endometrial receptivity, thereby increasing the risk of RSA [325, 326]. Additionally, research indicates that individuals with the KIR AA genotype may be more susceptible to CE, characterised by significantly reduced levels of endometrial cytokines TNF‐α and IL‐1β, which may further compromise pregnancy outcomes [327].

EMs is a heterogeneous, inflammatory and oestrogen‐dependent gynaecological disease characterised by the abnormal presence and growth of endometrial tissue outside the uterine cavity. Approximately 5%–10% of women of reproductive age are affected, often experiencing chronic pelvic pain and reduced fertility [328]. Two main hypotheses exist regarding the mechanisms underlying EMs. One study suggests that women with EMs may have immune system deficiencies. Studies have shown significantly reduced cytotoxicity of pNK cells in women with a history of EMs [329], although this finding remains controversial [330].

Experiments indicate that co‐culturing ESCs with macrophages results in a marked downregulation of NK cell CD16, NKG2D, perforin and IFN‐γ expression, activity and cytotoxicity, alongside increased secretion of IL‐1β, IL‐10 and TGF‐β. These changes may contribute to immune evasion of ectopic fragments, promoting the onset and progression of EMs [331]. Conversely, elevated TGF‐β1 expression may downregulate NKG2D expression on NK cells, further reducing their cytotoxicity [332]. Additionally, patients with EMs exhibit significantly lower expression of NK cell subsets CD56^+^/NKp46^+^ and CD56^dim^/NKp46^+^ in peritoneal fluid compared to non‐EMs individuals [333].

Elevated IL‐6 levels in the peritoneal fluid of patients with EMs reduce NK cell cytolytic activity by modulating Src homology region 2‐containing protein tyrosine phosphatase‐2 and downregulating granzyme B and perforin expression, thereby impairing NK cell function [334]. Increased levels of NKG2D ligands in the peritoneal fluid of patients with EMs further compromise NK cell function, although the exact mechanisms remain to be elucidated [335]. Moreover, in patients with EMs, the inhibitory receptor NKG2A and its ligand HLA‐E are highly expressed in the peritoneal fluid, with HLA‐E exhibiting resistance to NK cell‐mediated lysis [336].

Compared to healthy women, patients with EMs exhibit significantly lower levels of stem cell factor (SCF) in their endometrium, impairing the maturation of uNK cells and potentially contributing to EMs‐associated infertility [337]. CD16^−^ NK cells, also known as FCGR3‐ cells, undergo differentiation that markedly reduces their cytotoxic activity, resulting in an immunosuppressive environment in the peritoneal cavity of patients with EMs. Mechanistically, the hypoxic microenvironment in EMs destabilises DNA methyltransferase 1 (DNMT1) expression and reduces methylation of the prostacyclin (PGI2) synthase (PTGIS) promoter. This enhances PTGIS transcription and PGI2 biosynthesis, promoting CD16^−^ NK cell differentiation [338].

Moreover, autophagy in ESCs leads to STAT3 inactivation and downregulation of haematopoietic cellular kinases (HCKs). Low HCK signalling induces ESCs to secrete CXCL8 and IL‐23A, suppressing miR‐1185‐1‐3p in NK cells and increasing PTGS2 expression, which drives FCGR3‐ NK cell differentiation. This process diminishes the secretion of cytotoxic molecules, including KIR2DL1, KIR3DL1, NCR3, NCR2, IFN‐γ, perforin‐1 and granzyme B [339].

In patients with EMs, elevated levels of IL‐15 inhibit CD16 expression on NK cells, reducing granzyme B and IFN‐γ in CD16^+^ NK cells. Additionally, IL‐15 decreases NKG2D expression in CD56^dim^CD16^−^ NK cells and NKP44 expression in CD56^bright^ CD16^−^ NK cells. IL‐15 also promotes the growth and invasion of ESCs, aiding their immune evasion and advancing disease progression [340].

In some patients with EMs, a significant presence of CD200S^+^ NK cell subsets is observed in early pregnancy decidua. These CD56^+^ uNK cells counteract the immunosuppressive effects of CD200L [341]. Conversely, a higher prevalence of cytotoxic CD16^+^ uNK cells and/or NKp46^+^CD56^+^ cells may create an inflammatory environment during implantation or decidualisation, increasing infertility risks [342, 343].

Additionally, patients with EMs exhibit a lack of HLA‐G expression in endometrial tissues and stromal cells, reducing immune suppression and promoting inflammation, adversely affecting embryo survival in early pregnancy [344]. A higher incidence of HLA‐C in patients with EMs compared to controls has also been observed, interacting with killer inhibitory receptors on NK cells and further reducing their cytotoxic activity [345].

Protopanaxadiol (PPD), an aglycone of ginsenosides, exhibits various biological functions and medicinal value. PPD has been shown to downregulate cytotoxicity‐related molecules in dNK cells, such as NKp30 and CD16, while upregulating proliferation markers like Ki67, VEGF, TGF‐β and CXCL10. These effects promote NK cell proliferation, reduce cytotoxicity, enhance angiogenesis and support maternal‐foetal immune tolerance, preventing miscarriage in pregnant EMs mice [346]. However, this mechanism remains controversial. Other studies suggest that PPD activates NK cell receptors NKp30 and NKp46, increases IFN‐γ expression and reduces IL‐10 levels in NK cells, thereby decreasing ectopic lesion growth in mouse EMs models [347].

Uterine injuries, such as miscarriage and hysteroscopic curettage, result in increased trophoblast invasion and enhanced placental vascular formation, accompanied by extensive changes in the immune cell profile at the maternal‐foetal interface. Following uterine injury, the proportion of dNK cells is significantly reduced, whereas the expression of TNF‐α and IL‐4 in the decidua is markedly upregulated, and IFN‐γ and IL‐10 levels are notably downregulated. In the placenta, the expression of MMP2, MMP3, MMP9 and dedicator of cytokinesis 4 is significantly elevated, whereas the serum levels of anti‐angiogenic factors are markedly diminished. This immune imbalance may cause abnormal trophoblast invasion and excessive placental vascular formation, adversely affecting pregnancy outcomes [348].

Adenomyosis significantly impairs fertility, aligning with the local and systemic immune changes observed during implantation. In patients with adenomyosis, the proportion of macrophages, NK cells and DCs in the uterus significantly decreases during the implantation period. This decrease is accompanied by reduced expression of VEGF, PLGF, platelet endothelial cell adhesion molecule, and IGF‐2, resulting in pregnancy loss [8].

In patients with ovulatory dysfunction, including polycystic ovary syndrome and primary ovarian insufficiency, immature CD8^+^ T cells and effector memory CD4^+^ T cells are significantly reduced, whereas circulating NK cells and regulatory NK cells are increased. Elevated NK cell levels in the ovaries may exert cytotoxic effects on granulosa cells, disrupting normal follicular development and exacerbating ovulatory dysfunction [349]. Weakened HLA‐KIR interactions between the NK1 and NK2 subsets and non‐senescent stromal cell subsets in decidual tissue contribute to missed miscarriages. Additionally, the markers deiodinase, iodothyronine and DIO2 in senescent stromal cells serve as potential risk indicators for chromosomal abnormalities in embryos, leading to missed miscarriages. These markers aid in risk assessment for missed miscarriages and provide guidance for the clinical diagnosis and management of recurrent miscarriages [350].

### 4.9 Hypoxic Microenvironment

The hypoxic microenvironment plays a complex role at the maternal‐foetal interface. Although NK cell exhaustion reduces the delivery of pro‐angiogenic factors and delays uterine spiral artery development, it does not completely halt their formation. This is due to decreased oxygen tension in the placenta, which stabilises hypoxia‐inducible factor 1α (HIF‐1α). HIF‐1α forms a heterodimer with HIF‐1β to regulate downstream gene expression, redirecting trophoblast differentiation towards an invasive phenotype. This process allows trophoblast cells to replace the endothelium of the uterine spiral arteries, extending the depth of the placental vascular bed and accelerating vascular remodelling, thereby ensuring proper placental formation and maintaining pregnancy [351]. Hypoxia‐induced glycolysis enhances cellular adaptation to low‐oxygen environments, maintaining rapid growth and invasive potential [352], while suppressing mTORC1 activity. In patients with RSA, inhibition of mTORC1 signalling in dNK cells reduces glycolysis and oxidative phosphorylation, leading to decreased production of IFN‐γ and TNF‐α, impaired CD107a‐dependent degranulation, and weakened dNK cell proliferation [353]. Additionally, epithelial membrane protein 2 (EMP2), highly expressed in secretory endometrium and trophoectoderm cells, promotes capillary formation. However, EMP2 deficiency results in failed placental angiogenesis and placental hypoxia, which further increases uNK cell recruitment and retention, enhancing HIF‐1α expression [354, 355]. The hypoxic microenvironment may be both a cause and a pathological outcome of RSA and RIF. Elucidating its mechanisms, particularly in relation to HIF‐1α signalling, immune regulation and oxidative stress, holds promise for developing personalised therapeutic interventions for patients. This will be a key direction for future research and clinical applications.

### 4.10 Other Factors

The role of miRNAs in RSA and RIF has become a focus of research. miRNAs are non‐coding RNA molecules, approximately 22 nucleotides in length, that regulate gene expression by binding to target mRNAs. They are involved in various biological processes, such as cell proliferation, differentiation, apoptosis and immune responses [356, 357, 358]. In RSA and RIF, miRNAs may significantly influence miscarriage and implantation failure by modulating immune responses, embryonic development and the endometrial environment [359]. Specific miRNAs exhibit unique expression patterns. For example, miR‐10b and miR‐214 are exclusively expressed in dNK cells, whereas miR‐200a‐3p is only expressed in pNK cells. Compared with pNK cells, dNK cells show upregulation of miR‐130b‐3p, miR‐125a‐5p, miR‐212‐3p and miR‐454 and downregulation of miR‐210‐3p and miR‐132 [360]. These miRNA expression profiles in dNK and pNK cells provide potential molecular markers for personalised risk assessment of RSA and RIF [357, 361]. Additionally, post‐translational modifications, such as glycosylation [362, 363] and methylation [364, 365] play critical roles in regulating the function and stability of proteins, nucleic acids and other biomolecules. Further research on these modifications and their mechanisms may enhance our understanding of RSA and RIF pathophysiology and offer novel insights into disease diagnosis and treatment.

## 5 Treatment of RSA and RIF

Treatment of RSA and RIF presents several challenges. First, the lack of a unified diagnostic standard makes accurately identifying the underlying causes difficult. Second, the treatment methods are diverse, and their effectiveness varies, with many patients showing poor responses to conventional treatments. Additionally, the complexity of immune regulation and embryo implantation complicates the development of effective treatment strategies, particularly for issues such as immune tolerance and angiogenesis. Finally, while some treatments, such as hormone therapy and immunosuppressive therapy, are used, their long‐term efficacy and safety require further validation. A summary of clinical trials for RSA and RIF is presented in Table 1.

**TABLE 1:** Clinical trials targeting RSA or RIF.

Intervention/treatment	Target	Treatment subject	Status	Clinical trial	Age (years)	Enrollment (actual)	Intervention/treatment
IVIG	pNK cells	RIF	Phase 2	NCT03174964	20–41	50	400 mg/kg of IVIG 2 days before ET
IVIG	—	RSA	—	NCT00606905	18–45	82	500 mg/kg, infusions every 4 weeks until 18–20 weeks of gestation
IVIG	pNK cells	RSA	Phase 2	NCT03174951	18–41	50	Intravenous 400 mg/kg, every 4 weeks through 32 weeks
IVIG (GB‐0998)	—	RSA	Phase 3	NCT02184741	≤ 41	99	400 mg/kg once daily for 5 days until 6 weeks and 6 days of gestation
IVIG	—	RSA	Phase 3	NCT00722475	18–40	82	Intravenous infusions, 25–35 g each time, 4–15 weeks gestation
Aspirin, LMWH, IVIG, Prednisone	—	Thrombophilia with RSA	Phase 4	NCT02990403	18–50	500	Aspirin, 75–100 mg/day, bid; LMWH 4100 IU/day, hypodermic injection; IVIG 300 mg/kg/2 weeks, intravenous injection, prednisone, 5–10 mg/day
IVIG, Prednisolone	pNK cells	RSA after assisted reproductive technologies	Phase 2	NCT04701034	18–41 years (Adult)	74	IVIG, 0.3–48 mL/kg, intravenous injection, prednisolone, 5 mg before ET and 10 mg after ET
Intralipid	pNK cells	RSA undergoing IVF/ICSI cycle	Phase 4	NCT01788540	18–40	300	Intravenous infusion, 2 mL, repeated within 1 week of a positive pregnancy test and every 2 weeks until the end of the first trimester
Intralipid	pNK cells	RSA	Phase 1	NCT03132779	18–38	34	Intravenous infusions, 18 mg
Prednisolone	uNK cells	RSA around the time of embryo implantation	Phase 3	NCT03902912	18–40	84	Oral 10 mg/day for 7 days, 5 mg/day for 3 days, 2 mg/day for 3 days, 1 mg/day for 3 days
Intralipid	uNK cells	RIF undergoing IVF/ICSI cycle	Phase 1 Phase 2	NCT02865785	20–38	320	Intravenous infusions, intralipid 20%
Intralipid	—	RIF undergoing ICSI	Phase 2 Phase 3	NCT01540591	20–40	200	IV infusion of intralipid 20% between Days 4 and 9 of ovarian stimulation and another dose when pregnant within 1 week of a positive pregnancy test
Intralipid	—	RIF undergoing IVF/ICSI cycle	Phase 4	NCT02487940	20–38 years (Adult)	100	Intralipid 20%, 9 mg/mL, intravenous infusion, followed by a final dose 2–3 weeks later when attending for a pregnancy scan
Intralipid	pNK cells	RIF undergoing ICSI cycle	Phase 3	NCT01916798	35–40	200	IV infusion of 250 mL of intralipid 20% solution on the day of ovum collection and another dose on the day of embryo transfer
Intralipid		RIF undergoing ICSI cycle	Phase 2	NCT03374163	21–42	142	Intravenous infusions, intralipid 20%
Lymphocyte immunotherapy	—	RSA and RIF	Phase 1 Phase 2	NCT03081325	20–40	292	Peripheral venous blood drawn from the husbands of patients was resuspended and administered intradermally three times at 3‐week intervals. Once conception occurred, two rounds of treatment at 8‐week intervals were administered
LMWH	—	RIF undergoing IVF/ICSI cycle	Phase 4	NCT02607319	18–38	165	3500 IU anti Xa/0.2 mL solution for injection in pre‐filled syringes
Aspirin	—	RSA	—	NCT02823743	≤ 39	400	75 mg orally daily from gestational weeks 7–35
LMWH, Aspirin	—	RSA with antiphospholipid syndrome	Phase 2	NCT01051778	19–37	60	Enoxaparin 40 mg plus low‐dose aspirin
Vitamin D	—	RSA	—	NCT06002035	18–45	1421	30 drops (relative to 1 mL) once a day
Mesenchymal stem cells	—	RSA	Phase 1 Phase 2	NCT05520112	18–49	20	Received standard treatment and autologous mesenchymal stem cells
Antibiotics, Probiotics	—	RSA	Phase 2	NCT03401918	18–45	41	Oral antibiotics and vaginal probiotics
Rapamycin	—	RIF	Phase 2	NCT03161340	20–41	121	Received rapamycin 2 days before IVF until 15 days after IVF
Sildenafil citrate	uNK cells	RSA	Phase 1	NCT03766594	20–35	90	25 mg tablets four times daily for 24 days preconceptionally, commencing on the first day of the last period
Aerobic cycling training	Endometrial NK cells	RSA	—	NCT06007560	≤ 40	30	50%–60% of VO2 max for 1 h twice (Weeks 1–6) or three times (Weeks 7–12) per week

### 5.1 Immune Therapy

Intravenous immunoglobulin (IVIG) has been applied in immune therapy for RSA and RIF, primarily by modulating the immune system to improve maternal‐foetal immune tolerance and increase pregnancy success rates [366, 367]. Studies have shown that IVIG treatment significantly reduces the percentage and activity of pNK cells [368, 369, 370, 371], thereby reducing their cytotoxic effects on the embryo [372, 373, 374, 375, 376]. Additionally, IVIG administration lowers the frequency of Th1 lymphocytes, transcription factor expression and cytokine levels while increasing the Th2 response, enhancing pregnancy success rates [377]. However, some patients may require individualised treatment [378], as immune resistance can occur in those who fail IVIG therapy [379]. Understanding the mechanisms underlying this resistance is crucial for overcoming it. Although IVIG shows promise as an immune‐modulating therapy for RSA and RIF, its specific molecular mechanisms require further investigation.

Intralipid, an intravenous lipid emulsion initially used for nutritional support in critically ill patients, has recently gained attention for its potential immunoregulatory functions in treating immune‐related RSA and RIF. Intralipid reduces NK cell cytotoxicity [380, 381], decreases levels of TWEAK and its receptor FN‐14 [382], and minimises attacks on embryonic and placental cells, thereby protecting embryo development. Lipid emulsions may serve as an alternative to IVIG, likely by regulating NK cell function and promoting trophoblast invasion [383].

Prednisolone, a corticosteroid with potent immunosuppressive and anti‐inflammatory effects, plays a key role in treating immune‐related RSA and RIF. For instance, a case report documented a woman with 19 miscarriages successfully delivering a healthy baby after prednisolone treatment [384]. Research indicates that prednisolone inhibits NK cell cytotoxicity [385, 386, 387], functioning similarly to IVIG. Additional treatments, such as intrauterine infusions of dexamethasone, granulocyte CSF (G‐CSF) [388, 389] and the TNF‐α antagonist etanercept [390], can reduce uNK cells and improve endometrial receptivity, facilitating embryo implantation [391]. A systematic review and meta‐analysis revealed that IVIG, prednisolone and intralipid therapies improve live birth outcomes. However, large‐scale randomised controlled trials are needed to validate these benefits in specific female subgroups [392].

Lymphocyte immunotherapy (LIT) is another treatment used for immune‐related RSA and RIF. By regulating maternal‐foetal immune tolerance, LIT can improve pregnancy success rates, especially in patients with evident immune abnormalities. LIT has been associated with reduced expression of miR‐326a and miR‐155, and increased expression of miR‐146a and miR‐10a, resulting in reduced inflammatory cytokines (e.g., IL‐17, IFN‐γ and TNF‐α) and increased anti‐inflammatory cytokines (e.g., IL‐4, IL‐10 and TGF‐β). This reduces NK cell cytotoxicity [393]. An emerging immune therapeutic strategy involves in vitro expansion of healthy dNK cells and their reintroduction into the body, demonstrating significant therapeutic potential [394].

### 5.2 Anticoagulant Therapy

Anticoagulant therapy is crucial for treating RSA and RIF because blood‐clotting abnormalities and microcirculatory disorders are key factors in their pathogenesis. aPLs, such as anticardiolipin and anti‐β2‐glycoprotein I antibodies, along with immune abnormalities like high NK cell activity or Th1/Th2 imbalance, can activate the coagulation system, leading to increased platelet aggregation, hypercoagulability, uterine microcirculatory disturbances and embryo implantation failure. Treatment with low molecular weight heparin (LMWH) reduces the uterine radial artery resistance index and improves pregnancy outcomes in women with RSA [395].

The combination of LMWH and low‐dose aspirin prevents thrombosis, improves placental blood flow, and increases pregnancy success rates [396]. For patients with unexplained recurrent miscarriage, adding prednisone to heparin and low‐dose aspirin may be beneficial due to steroids' suppression of peripheral CD16 NK cell concentrations [397]. Combining anticoagulant therapy with immunosuppressive agents like prednisone and enoxaparin [398] or IVIG and low‐dose aspirin [399] forms a comprehensive treatment strategy for patients with RSA and RIF, particularly those with hypercoagulable states and immune abnormalities. This integrated approach addresses immune imbalances and microcirculatory disorders at the maternal‐foetal interface, significantly improving pregnancy success rates.

However, combination therapies must be personalised and closely monitored to ensure efficacy and safety. This approach offers renewed hope for patients facing pregnancy challenges.

### 5.3 Vitamin D Therapy

Vitamin D deficiency is common among women with RSA, increasing their risk of autoimmune and cellular immune abnormalities. Recent studies have emphasised the critical role of vitamin D in regulating maternal‐foetal immune tolerance, improving endometrial receptivity, and supporting embryo implantation. Vitamin D deficiency (< 30 ng/mL) is an independent risk factor for hyperhomocysteinemia and elevated NK cell cytotoxicity [400]. In women with low vitamin D levels, NK cell cytotoxicity is significantly higher than in women with normal levels. These patients also show increased serum levels of IL‐2, TNF‐α, IFN‐γ and IL‐6, along with decreased levels of IL‐4 and IL‐10.

Vitamin D supplementation, both in vitro and in vivo, has been shown to significantly reduce NK cell levels of IL‐2, IFN‐γ and TNF‐α, while increasing levels of IL‐4, IL‐10, IL‐1β, VEGF and G‐CSF, thereby improving pregnancy outcomes [401, 402, 403, 404]. The active form of vitamin D, 1,25‐dihydroxyvitamin D3 (1,25(OH)_2_D_3_), is a potent immunomodulatory steroid that regulates both innate and adaptive immunity. Its immune‐regulatory effects are similar to those of IL‐10 and can dose‐dependently reduce natural cytotoxicity, making it a potential treatment for RSA [405, 406, 407]. This active form inhibits the expression of GM‐CSF, TNF‐α and IL‐6 in dNK cells while promoting the secretion of antimicrobial peptides such as cathelicidin [408, 409]. However, other studies indicate that 1,25(OH)_2_D_3_ has no significant overall impact on gene expression in peripheral or uNK cells [410].

Patients with RSA with low vitamin D levels also exhibit significantly reduced HLA‐G protein and mRNA levels, which compromise immune suppression and affect early embryo survival [411]. As a safe and effective immunomodulator, vitamin D supplementation plays an essential role in treating RSA and RIF. It offers a new avenue for improving pregnancy outcomes by regulating immune tolerance, enhancing endometrial receptivity and exerting anti‐inflammatory effects. However, appropriate dosing and serum monitoring are crucial to optimising treatment efficacy.

### 5.4 Stem Cell Therapy

Stem cells, including haematopoietic stem cells (HSCs) and mesenchymal stem cells (MSCs), hold significant promise in treating RSA and RIF owing to their immune‐regulatory and anti‐inflammatory properties. These cells have been extensively studied for their potential to regulate maternal–fetal immune tolerance and improve the uterine microenvironment.

HSCs can differentiate into various blood and immune cells, including NK cells. Decidual tissue‐derived HSCs, identified as Lin^−^ CD34^+^ CD45^+^ cells, can be cultured with IL‐15 and SCFs to generate NK cells [55, 412]. CYNK‐001, a product derived from human placenta‐derived HSCs, expresses high levels of activating receptors and demonstrates strong cytotoxic activity. It has shown anti‐inflammatory and immune‐regulatory effects against the H1N1 influenza virus, suggesting potential applications in targeting infected trophoblasts [413]. However, the therapeutic benefits of HSCs for RSA and RIF remain uncertain. Immunosuppressive agents, such as mycophenolate mofetil and cyclosporine A, may be needed post‐HSC transplantation, potentially impairing NK cell functions [414].

MSCs, derived from mesenchymal tissues, have the potential to differentiate into non‐haematopoietic cells. In women with RSA, weakened interactions between NK cells and other cells reduce tolerance‐related biomarkers, such as osteopontin, in NK cells. Treatment with Wharton's jelly‐derived MSCs has been shown to improve the immune‐tolerant microenvironment at the maternal‐foetal interface, supporting pregnancy maintenance [415]. MSCs inhibit uNK cell secretion of IFN‐γ and promote IL‐4 and IL‐10 expression, shifting NK cells from an inflammatory to a tolerant state [416]. Activin‐A secreted by human umbilical MSCs strongly inhibits IFN‐γ production in NK cells by reducing STAT4 phosphorylation, NF‐κB activity and T‐bet activation [417]. However, MSCs are often rapidly cleared by the host's innate immune cells, limiting their therapeutic applications [418]. Further clinical research is required to fully explore and optimise stem cell therapy for RSA and RIF.

### 5.5 Traditional Chinese Medicine

Traditional Chinese Medicine (TCM) offers promising potential for treating RSA and RIF with fewer side effects compared to Western medications. TCM primarily improves pregnancy success rates by regulating immune function, enhancing the uterine environment and supporting overall health.

In LPS‐treated mice, the number of dNK cells and IL‐2 levels significantly increase, whereas a herbal formula composed of *Scutellaria baicalensis* and *Atractylodes macrocephala* effectively reduces dNK cell numbers and IL‐2 levels, thereby preventing miscarriage [419]. Additionally, the Bu‐Shen‐Yi‐Qi formula (comprising Dangshen, Baizhu, Tusizi, Baishuo, Sangjisheng, Duzhong, Tiaohuangqin and Sugeng) upregulates IDO expression in trophoblast cells, reducing NK cell cytotoxicity [420].

Traditional cupping therapy has also demonstrated efficacy in reducing NK cell numbers, activity and cytotoxicity [421]. Integrating Chinese and Western medicine may provide synergistic effects, improving clinical outcomes for patients with RSA and RIF. Chinese herbs show potential as adjunctive treatments, improving immune tolerance, modulating the uterine environment and enhancing overall health. High‐quality clinical studies are necessary to elucidate the mechanisms and therapeutic effects of TCM, providing a foundation for precise and individualised treatment approaches.

### 5.6 Antimicrobial Therapy

Antimicrobial therapy is primarily used in the treatment of RSA and RIF to address potential reproductive tract infections or CE, which may interfere with embryo implantation or lead to pregnancy failure. Antibiotics inhibit bacterial growth or kill bacteria to achieve therapeutic effects. However, the use of antibiotics during pregnancy carries risks of foetal development disruption and drug toxicity. To mitigate these risks, Crespo et al. infused antimicrobial peptide granulysin (GNLY) into extravillous trophoblasts of the placenta through nanotubes, successfully killing intracellular *Listeria monocytogenes* without killing the trophoblast [291]. Zinc supplementation has also been observed to enhance immune responses during pregnancy and improve the host's ability to combat infections, such as *Trypanosoma cruzi*. Zinc has been shown to boost immune responses even before pregnancy [422]. Additionally, killed streptococcal preparation (KSP), an immunomodulator derived from inactivated streptococcal cultures, is used to regulate immune responses. KSP has been found to maintain pregnancy by improving immune tolerance and regulating immune functions, offering a simple and safe alternative therapy for unexplained RSA [423]. Therefore, further exploration of the regulatory role of NK cells and their molecular mechanisms in these studies may provide new potential strategies for the treatment of RSA and RIF. It is important to note that the uterus and vagina have specific microbiota, and maintaining their balance is crucial for successful pregnancy outcomes. Antimicrobial therapy may disrupt this balance, potentially leading to secondary infections or inflammation.

### 5.7 Nanomaterial and Hydrogel Therapy

Nanomaterials, substances measuring 1–100 nm, possess distinct physical and chemical properties owing to their small particle size and high surface area, making them highly effective for targeted therapies. Drugs can be precisely delivered using nanoparticles, improving therapeutic outcomes. For instance, the GC‐Exo‐CD16Ab system utilises purified exosome carriers derived from human umbilical MSCs to load GCs and modify the antibody CD16Ab. This system exhibits excellent biocompatibility and efficiently targets CD16^+^ dNK cells and macrophages, inhibiting NK cell cytotoxicity and M1 macrophage polarisation. It modulates the decidual microenvironment, improves placental and foetal morphology and significantly reduces miscarriage risks in mouse models, offering a new strategy for regulating the pregnancy microenvironment [424].

Despite their promise, nanomaterials also pose toxic side effects. For example, exposure to titanium dioxide nanoparticles during pregnancy significantly impairs placental growth and development, likely due to dysregulated angiogenesis, proliferation and apoptosis. Pregnant women should exercise caution when handling nanomaterials [425].

Hydrogels, composed of hydrophilic polymer networks capable of absorbing large amounts of water, are widely used in drug delivery and tissue repair. Recent studies highlight their potential in sustaining therapeutic effects. For example, exosomes derived from villi carrying miR‐29a‐3p inhibit IFN‐γ production in dNK cells by binding to the 3′ UTR of their mRNA. Hyaluronate gel extends the residence time of vEXOs in the uterine cavity, enabling sustained release. Engineered exosomes loaded with miR‐29a‐3p significantly reduce embryo resorption rates in RSA mice without systemic toxicity, showing promising safety and therapeutic potential [9].

The integration of nanotechnology and hydrogels has led to the development of nanocomposite hydrogels, combining the benefits of nanomaterials and hydrogels. These hybrid systems show great potential in drug delivery, tumour therapy and tissue repair.

In addition to the aforementioned therapies, several biologics have been explored for treating RSA and RIF, including sildenafil citrate [426, 427, 428], synthetic preimplantation factor [429], low‐dose rapamycin [201], collagenase‐1 [430] and dietary L‐proline supplementation during pregnancy [431]. These biologics function by inhibiting NK cell cytotoxicity, reducing pro‐inflammatory cytokines such as TNF‐α, IL‐1α, IL‐6, IL‐23 and IFN‐γ, while increasing levels of immunoregulatory factors like TGF‐β, IL‐10, IL‐17, GM‐CSF and VEGF. These changes enhance immune tolerance and improve pregnancy outcomes. However, further validation through extensive research is required to confirm their safety and reliability before their widespread clinical use.

## 6 Conclusions and Perspectives

Over the past five decades, significant progress has been made in understanding the role of NK cells in pregnancy failures. uNK cells exhibit a dual role in pregnancy, regulating placental angiogenesis and immune tolerance by secreting factors such as IFN‐γ and VEGF. However, their abnormal activation or dysfunction can lead to placental insufficiency and immune‐mediated pregnancy failure. These findings have provided the groundwork for developing therapeutic strategies to improve pregnancy outcomes.

Despite advancements, the multifactorial and complex nature of the uterine microenvironment means that the precise mechanisms underlying NK cell function remain unclear. Developing ideal in vitro and in vivo models to simulate the uterine microenvironment during pregnancy is critical for further research. Current in vitro models, such as three‐dimensional organ culture systems [432] and two‐chamber microphysiological systems [433], cannot fully replicate the uterine complexity. There are significant differences in the characteristics of human and rodent decidual NK (dNK) cells, which limit our ability to study the in vivo functions of human dNK cells using rodent models. To overcome this limitation, researchers have developed a mouse model in which human dNK cells are adoptively transferred into pregnant NOG (NOD/Shi‐scid/IL‐2Rγ^null^) mice. The results showed that these cells primarily homed to the uterus of recipient mice. This model provides a valuable tool for investigating the characteristics of human dNK cells and their relationship with pregnancy outcomes [434]. Additionally, the roles of other immune cells, such as B cells, macrophages and Tregs, in influencing uNK cell function remain insufficiently understood [11, 435, 436].

Reliable large‐scale data studies are also lacking, particularly regarding racial differences in uNK cell function [437]. Furthermore, existing therapies—such as immunotherapy, anticoagulant therapy, vitamin D supplementation, stem cell therapy, TCM, antimicrobial therapy and nanomaterial‐based hydrogel treatments—show promise despite challenges, including long‐term immune effects on the mother and foetus.

Future research should prioritise elucidating the molecular mechanisms of uNK cells to develop more targeted therapeutic strategies. Systematic evaluation of existing and emerging treatments is essential to provide personalised, effective and safe solutions for pregnancy failure‐related conditions.

## Author Contributions

Defeng Guan, Zhou Chen, Lifei Li and Xia Huang conceived the review. Defeng Guan, Zhou Chen, Yuhua Zhang, Wenjie Sun, Lifei Li and Xia Huang undertook the initial research. Defeng Guan and Zhou Chen were involved in writing, Xia Huang and Lifei Li reviewed the manuscript, and all authors contributed to the final version. Zhou Chen contributed equally to this work and should be considered co‐first author, Lifei Li should be considered as a co‐corresponding author. All authors have read and approved the article.

## Ethics Statement

The authors have nothing to report.

## Conflicts of Interest

The authors declare no conflicts of interest.

## Data Availability

The authors have nothing to report.
